# Electronic Structure Reorganization in MPS_3_ via d‐Shell‐Selective Alkali Metal Doping

**DOI:** 10.1002/advs.202510675

**Published:** 2026-03-24

**Authors:** Jonah Elias Nitschke, Preeti Bhumla, Till Willershausen, Patrick Merisescu, David Maximilian Janas, Lasse Sternemann, Michael Gutnikov, Karl Schiller, Valentin Mischke, Michele Capra, Mira Sophie Arndt, Silvana Botti, Mirko Cinchetti

**Affiliations:** ^1^ TU Dortmund University Dortmund Germany; ^2^ Research Center Future Energy Materials and Systems of the University Alliance Ruhr and Interdisciplinary Centre for Advanced Materials Simulation, Faculty of Physics and Astronomy Ruhr University Bochum Bochum Germany; ^3^ University of Bath Bath UK

**Keywords:** 2D materials, electron doping, MPS_3_

## Abstract

Semiconducting two‐dimensional (2D) antiferromagnetic (AFM) transition‐metal thiophosphates (MPS_3_) offer promising opportunities for spintronic applications due to their highly tunable electronic properties. While alloying and intercalation have been shown to modulate ground states, the role of d‐shell filling in governing these transitions remains insufficiently understood. Here, we investigate electron doping effects in MPS_3_ using angle‐resolved photoemission spectroscopy (ARPES), X‐ray photoelectron spectroscopy (XPS), and density functional theory (DFT+U). Lithium and cesium deposition are employed to induce doping across different MPS_3_ compounds. We identify two distinct doping mechanisms: in MnPS_3_, electrons are primarily donated to the P_2_S_6_ ligand clusters, with negligible Mn 2p core‐level shifts and no major changes in the valence band. In contrast, FePS_3_, CoPS_3_, and NiPS_3_ exhibit clear reductions in transition‐metal oxidation states, with a ∼1.0 eV reduction in spin‐orbit splitting for Co upon doping. ARPES on CoPS_3_ reveals a ∼400 meV shift of Co‐derived bands toward higher binding energies and new dispersive states up to 1 eV above the valence band maximum, indicating metallic behavior. These results establish a direct correlation between d‐shell filling and doping response, highlighting alkali metal doping as a tunable route to tailor the electronic and magnetic properties of 2D AFM semiconductors for spintronic applications.

## Graphical abstract

Alkali metal doping of layered MPS_3_ antiferromagnets reveals d‐shell‐selective responses. While MnPS_3_ resists charge transfer due to its half‐filled 3d^5^ shell, FePS_3_ and NiPS_3_ behave similarly to CoPS_3_ by accommodating extra d‐electrons. Yet only CoPS_3_, with its least stable d‐shell, undergoes pronounced band restructuring and a semiconducting‐to‐metallic transition.

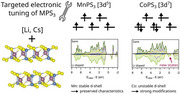

## 1 Introduction

Van der Waals (vdW) materials with intrinsic magnetic order have gained significant attention due to their potential for novel quantum technology applications [1, 2]. Their two‐dimensional (2D) nature and ease of exfoliation [3, 4] make them extremely versatile, enabling a variety of tuning strategies, including external gating [5, 6], alloying [7], intercalation [8, 9], and engineered stacking of different layers to create designed interfaces that exploit moiré potentials and proximity effects [2].

**FIGURE 1:**
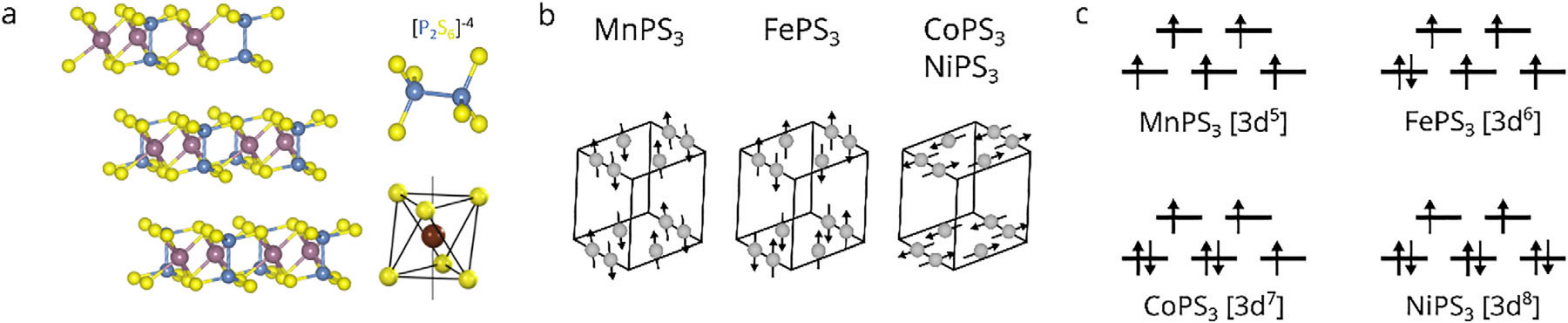
Structural, magnetic, and electronic properties of MPS_3_ compounds. (a) Crystal structure of MPS_3_ showing the shifted stacking parallel to the cleavage plane on the left side. The right side displays the structure of the P_2_S_6_ clusters along with the octahedral environment around the M ions by the surrounding S atoms. (b) Varying antiferromagnetic spin structures and (c) d‐orbital electronic configuration of the d‐orbitals of the different MPS_3_ materials.

Among 2D vdW magnets, antiferromagnetic (AFM) compounds are gaining much attention due to their intrinsic insensitivity to external magnetic fields and ultra‐fast spin dynamics in the terahertz regime [10]. A particularly interesting family of 2D AFMs are the transition‐metal thiophosphates (MPS_3_), whose magnetic structure arises from a honeycomb lattice of transition metal ions (M^2+^ = Fe^2+^, Ni^2+^, Co^2+^, Mn^2+^) within the crystal field of surrounding [P_2_S_6_]^4−^ bipyramidal units [11, 12, 13, 14, 15] (Figure 1a,b). In these systems, magnetism originates from competing exchange interactions, primarily between transition‐metal cations via direct and superexchange pathways [16]. These interactions create a delicate balance, which makes MPS_3_ compounds highly tunable through internal modifications, such as alloying and intercalation, as well as external stimuli like strain and electrostatic gating [17, 18].

Recent efforts have demonstrated that alloying in MPS_3_, through the substitution of transition‐metal cations, can significantly alter exchange interactions [7, 19, 20]. This leads to changes in the magnetic ground state, including transitions between Néel, zigzag, ferrimagnetic and spin‐glass orders [19, 21, 22]. Intercalation with molecular species or electrochemically inserted cations, on the other hand, can modify the balance between direct and superexchange interactions, inducing phase transitions from antiferromagnetic to ferromagnetic or ferrimagnetic states [8].

A common consequence of both alloying and intercalation is the alteration of d‐shell filling, either by introducing charge carriers or by substituting the transition‐metal species. Such modifications have a direct impact on magnetic exchange pathways and ordering, yet a comprehensive understanding of how d‐electron count governs the emergence of magnetic and electronic phases remains lacking. This gap in understanding limits the controlled design of MPS_3_‐based materials for future quantum technologies.

Here, we combine angle‐resolved photoemission spectroscopy (ARPES), X‐ray photoelectron spectroscopy (XPS), and density functional theory (DFT+U) calculations to investigate how electron doping via lithium and cesium deposition affects the electronic structure across the MPS_3_ family. Our systematic study reveals compound‐specific responses, with MnPS_3_ exhibiting markedly different behavior compared to FePS_3_, CoPS_3_, and NiPS_3_. We aim to understand the underlying mechanisms governing these differences and their implications for electronic and magnetic structure modification. In contrast to previous experimental and theoretical works on alkali metal intercalation [21, 23, 24, 25, 26, 27], we focus specifically on how electron doping affects the d‐shell filling of the M^2+^ ions (Figure 1c), and how alteration of the d‐shell filling influences the electronic structure of the compounds.

In the following we highlight MnPS_3_ and CoPS_3_ as two representative cases that exemplify the distinct doping responses observed, while detailed results for FePS_3_ and NiPS_3_ can be found in Supplementary Information. By employing XPS, we investigate the changes induced to the MPS_3_ core‐level state due to Lithium doping. This reveals two different doping mechanisms that either lead to a change of oxidation state of the M^2+^ ion (for M = Fe, Ni, Co) or a predominant electron localization on the P_2_S_6_ clusters (for M = Mn). Complementary ARPES measurements on the bare MPS_3_ surfaces are first used to benchmark our DFT+U calculations, before the investigation of the Li doped surface uncovers how the electron doping reshapes the valence band structure, most notably in CoPS_3_ with the least stable d‐shell configuration. These findings are corroborated by further cesium based measurements, revealing that the induced modifications scale with the doping level and are therefore highly tunable.

Together, these findings establish alkali metal doping as a powerful and selective strategy to modulate the electronic structure of 2D antiferromagnets. By directly linking d‐shell occupancy to band structure evolution and charge redistribution, our work offers a clear framework for tailoring the electronic and magnetic functionalities of MPS_3_ compounds, paving the way for their integration into next‐generation spintronic devices.

## 2 Results

### 2.1 XPS Study

**FIGURE 2:**
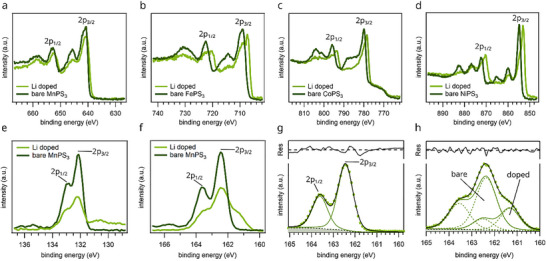
XPS spectra for M 2p, P 2p, and S 2p levels for the various MPS_3_ compounds before (dark green) and after lithium doping (bright green). (a—d) show XPS measurements on the M 2p states for the bare MPS_3_ surface as well as the lithium doped surface. For all spectra the contributions stemming from the M 2p_3/2_ and 2p_1/2_ orbitals are labelled. The remaining peaks are satellites associated with ligand‐to‐metal charge transfer. The shoulder visible in the Co 2p spectrum between 770 and 780 eV originates in the L_2_M_23_M_45_ Auger transition, which lays close to the Co 2p_3/2_ peak when using an Al K_α_ light source. (e) and (f) present XPS measurements on the P 2p and S 2p peaks for bare MnPS_3_ (dark green) and lithium doped MnPS_3_ (bright green), respectively. A fit of the S 2p spectra displayed in (g) and (h) indicates only one oxidation state for the bare MnPS_3_, while the necessity of two doublets to fit the lithium doped surface hints toward two different moieties of sulfur atoms (doped and undoped).

To assess how the electronic configuration of the transition metal ions evolves with electron doping, we investigate the M 2p core‐level spectra shown in Figure 2a–d. These spectra allow us to identify changes in oxidation state and multiplet structure arising from lithium doping. The data reveal two distinct response categories: one exemplified by MnPS_3_ and the other by Fe‐, Co‐, and NiPS_3_.

Starting with MnPS_3_ (Figure 2a), the undoped surface shows sharp spin‐orbit split 2p_3/2_ and 2p_1/2_ peaks, both exhibiting pronounced multiplet splitting due to interactions between core and valence electrons [28, 29, 30, 31, 32, 33, 34]. This structure is well reproduced using multiplet‐based fitting models from Gupta et al. [28, 29]. (see Figure S1). The 2p peaks are followed by well‐defined satellite features attributed to shake‐up processes [33, 35]. After lithium doping, the Mn 2p spectrum remains largely unchanged: no significant shift is observed in the main peaks, and only slight peak broadening and a reduction in satellite intensity occur. This suggests that Mn retains its 2+ oxidation state, in agreement with the computational results that will be presented later.

In contrast, Fe‐, Co‐, and NiPS_3_ (Figure 2b–d) show substantial modifications upon lithium doping. New peaks emerge at lower binding energies relative to the original 2p_3/2_ and 2p_1/2_ peaks, indicating the appearance of a new oxidation state rather than a uniform chemical shift. These changes evolve with lithium coverage (see Figure S2) and are accompanied by a reduction in spin‐orbit splitting (see Table S3). For example, CoPS_3_ shows a decrease in 2p peak splitting from 16 eV (bare surface) to 15 eV after doping, consistent with a transition from Co^2+^ in a high‐spin 3d^7^ (S = 3/2) configuration to a lower spin 3d^9^ (S = 1/2) state, corresponding to Co^0^. This interpretation is supported by comparison with previous studies of Co complexes by Frost et al. [36]. Similar, though less pronounced, reductions in spin–orbit splitting are also observed for NiPS_3_ and FePS_3_ (see Table S3).

Additionally, lithium doping alters the satellite structure of the M 2p spectra. For instance, NiPS_3_ originally shows distinct satellites ∼10 eV above the main peaks (labeled Sat2_3/2_ and Sat2_1/2_ in Table S4 and Section S5), which vanish upon doping. Such satellite features are associated with ligand‐to‐metal charge transfer and multiplet effects involving final‐state interactions [33]. Their disappearance hints toward a change in final‐state screening and electronic structure, further supporting a modified electronic configuration in the doped compounds. The remaining satellites shift in energy in accordance with the new spin‐orbit splitting values.

Besides the transition metal core levels, we also analyzed the XPS signals from the phosphorus and sulfur atoms, focusing on the P 2p and S 2p regions. For Fe‐, Co‐, and NiPS_3_, both peaks remain unchanged upon lithium doping, indicating that the ligand environment is largely unaffected (see Section S6). The same holds for the P 2p peak in MnPS_3_ (Figure 2e). However, the S 2p spectrum of MnPS_3_ exhibits a distinct transformation upon lithium doping. As shown in Figure 2f, the bare surface displays the characteristic spin‐orbit split 2p_3/2_ and 2p_1/2_ components, which are not fully resolved due to instrumental limitations. After lithium deposition, the overall intensity of the S 2p signal decreases, and an additional shoulder appears at lower binding energy. Figure 2g,h compares the S 2p spectra of the bare and Li‐doped MnPS_3_ surfaces. While the undoped sample displays a single doublet, the doped surface requires fitting with two distinct doublets. One matches the original S 2p position, while the other is shifted to lower binding energy, indicating the emergence of a second sulfur species. To summarize, the XPS data demonstrate that lithium deposition alters the oxidation state of the M^2^^+^ ions in Fe‐, Co‐, and NiPS_3_, as the additional charge is incorporated into the transition‐metal d orbitals, resulting in a transition from M^2^^+^ to a M^0^‐like configuration in CoPS_3_. In MnPS_3_ instead, the lithium‐derived electrons predominantly localize on the P_2_S_6_ ligand clusters rather than the transition metal center. This contrast underscores the critical role of the transition‐metal electronic configuration in determining the doping mechanism.

### 2.2 ARPES Study

**FIGURE 3:**
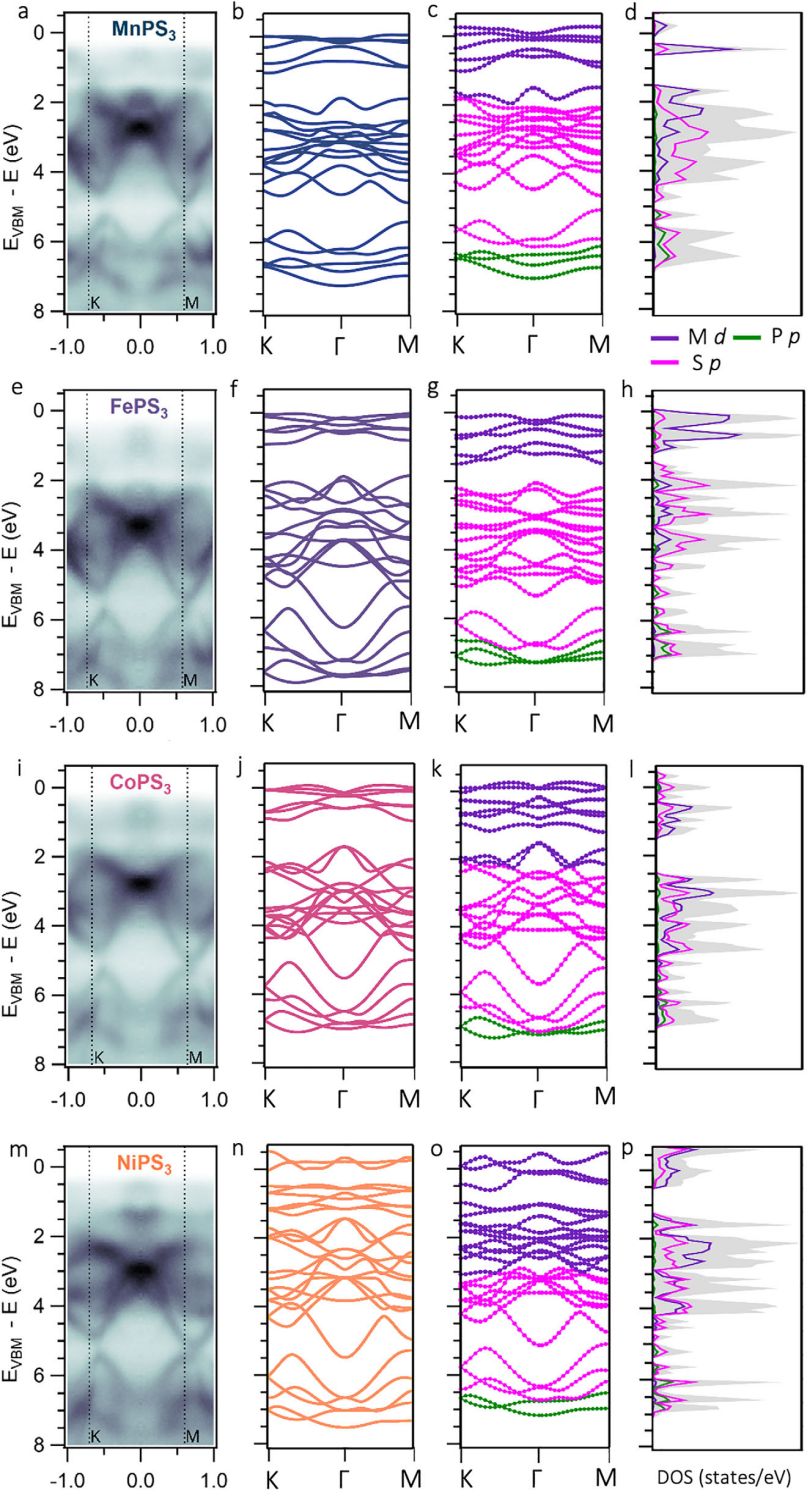
ARPES measurements and DFT+U calculations for different MPS_3_ compounds. Panels (a), (e), (i), and (m) show ARPES measurements of Mn‐, Fe‐, Co‐, and NiPS_3_, respectively, along the K–Γ–M cut, measured using a monochromatized He II lamp with a photon energy of 21.22 eV. Panels (b), (f), (j), and (n) show the corresponding DFT+U band structures for the nonmagnetic (NM) phase, while panels (c), (g), (k), and (o) show the orbital‐projected band structures for the paramagnetic (PM) phase along the same high‐symmetry path. Panels (d), (h), (l), and (p) display the density of states (DOS) in the PM phase. All these calculations are performed for monolayer systems.

We now investigate how the distinct electron doping behavior identified by XPS modifies the electronic band structure of the MPS_3_ compounds. To this end, we performed angle‐resolved photoemission spectroscopy (ARPES) measurements on all four systems. As a reference, we first characterized the pristine surfaces of MnPS_3_, FePS_3_, CoPS_3_, and NiPS_3_ to benchmark our DFT+U calculations and establish a basis for analyzing lithium‐induced changes. The corresponding ARPES spectra along the high‐symmetry path K–Γ–M are shown in Figure 3a,e,i, and m, respectively. To reduce the influence of potential effects from laser incidence angle and light polarization [37], all momentum maps were symmetrized according to the hexagonal lattice symmetry prior to extracting the band structure (see Figure S7 for details).

The second and third column in Figure 3 show, respectively, the calculated electronic band structures of the compounds in both the NM and PM phases, since they exist in the PM phase at room temperature [13, 37]. The measured band structures show good agreement with the DFT calculations using the PBE+U functional. Although the complexity of the electronic structure prevents a direct, band‐by‐band comparison between the experimental results and the theoretical calculations, the DFT+U approach successfully reproduces the experimentally observed gaps, which separate regions dominated by relatively flat, localized bands from those with more strongly dispersive character [11].

Furthermore, both the experimental data and the DFT+U results capture the characteristic band extrema and primary dispersive features, including a pronounced maximum of the band at approximately 5 eV at the M point, as well as a secondary maximum located slightly before the K point. In the PM band structures, the hybridization of M‐d and S‐p states results in slightly increased dispersion within the 0–2 eV range compared to the NM band structures. Importantly, however, the overall qualitative electronic structure remains largely unchanged across the compounds, which justifies our use of the NM calculations in the following analysis. A zoomed‐in view of the band structures in the 0–3 eV range is provided in Figure S8, while ARPES measurements of neighboring Brillouin zones are shown in Figure S9 to enable a more comprehensive comparison with theory, as some dispersive bands are only observable at larger parallel momenta due to photoemission matrix‐element effects. Additionally, to examine the k_z_ dispersion in our calculations, we plot the band structures at intermediate values of k_z_ = 0.0, 0.2, and 0.4/Å to illustrate the quasi‐2D nature of the electronic structure [13] (see Figure S10). The calculations show that the bands exhibit weak dispersion along k_z_, with variations smaller than ∼100 meV, which is comparable to the effective energy resolution achievable for 2D semiconductors with our ARPES setup [38].

In the following, we focus on the changes introduced by lithium doping in MnPS_3_ and CoPS_3_, which represent the two extremes in doping response within the MPS_3_ family. Before examining the valence band, we first assess whether lithium incorporation induces any structural changes in these compounds. Our DFT+U calculations show that adding Li causes no significant structural rearrangements. The layers expand by less than 1%, and bond lengths remain essentially unchanged, indicating Li^+^ can fit into the van der Waals gap without major distortion. While MnPS_3_ can host Li^+^ slightly more easily than NiPS_3_, the overall structural effects are minimal across all compounds [38, 39]. Thus, the electronic modifications reported below arise predominantly from charge doping rather than from lattice deformation. Note that we modeled doping by incorporating one Li or Cs atom in a 2 × 2 × 2 supercell, corresponding to an electron doping of approximately 0.12 e^−^ per Mn atom (∼12% per formula unit; one Li atom in an 80‐atom cell). Since the experimental doping concentration is difficult to quantify in our surface deposition experiments, we chose this concentration to balance computational feasibility with a realistic doping level. The same doping concentration was used for all compounds to allow for a qualitative comparison between different materials. The systematic shifts observed in both the XPS core levels and the ARPES valence bands with increasing alkali metal deposition time (Figures S2, S11, and S12) demonstrate that the electronic structure modifications scale with the doping level.

**FIGURE 4:**
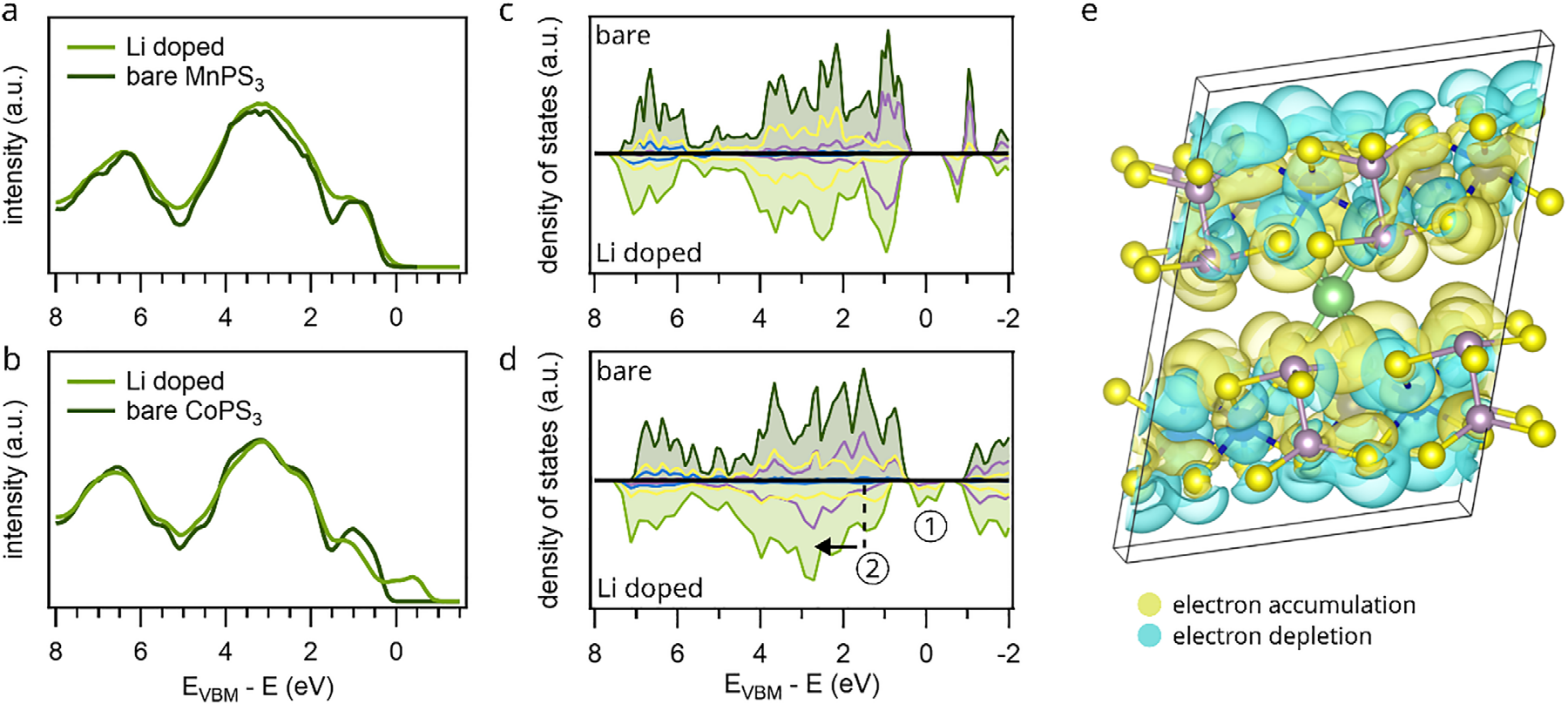
Changes in the valence band and work function after alkali metal deposition. (a), (b) display the EDC for Mn‐ and CoPS_3_ before (dark green) and after alkali metal deposition (light green), derived from our ARPES measurements by integration over the whole available momentum space. All spectra were obtained using the helium lamp at 21.22 eV photon energy. (c), (d) show the DOS of monolayers calculated by DFT+U in NM phase for the bare surface (top part) and doped surface (bottom part). The purple, yellow and blue lines correspond to the M, S and P ions, respectively. e) charge density difference distribution of the Li doped CoPS_3_, where the yellow and cyan regions represent electron accumulation and depletion, respectively.

Figure 4a,b shows the energy distribution curves (EDCs) for MnPS_3_ and CoPS_3_, respectively, before (dark green) and after (bright green) lithium deposition. In MnPS_3_, lithium doping induces only minor changes with a slight broadening of spectral features, whereas in CoPS_3_, a clear signal emerges above the original valence band maxiumum (VBM). Additionally, the peak closest to the VBM shifts to higher binding energy and appears only as a shoulder in the doped spectrum.

These experimental observations are supported by density of states (DOS) calculations. For MnPS_3_, lithium doping results in no significant redistribution of states, apart from overall spectral broadening (Figure 4c). The lack of energetic shifts in the Mn‐related states indicates that the Mn oxidation state remains unchanged, consistent with our XPS analysis. This stability can be attributed to the half‐filled 3d^5^ configuration of Mn^2+^, which is a particularly energetically stable electronic configuration.

In contrast, the calculated DOS of CoPS_3_ (Figure 4d) reveals substantial changes upon lithium doping. New states appear near the VBM (label 1), which arise predominantly from hybridized

Co 3d and S 3p orbitals and are shifted to lower energy in comparison to the bare surface due to Coulomb interaction with the additional electron donated by Li. Crucially, these states are not localized impurity states introduced by Li itself but rather reflect a reorganization of the CoPS_3_ electronic structure induced by doping. This reorganization in turn implies that lithium alters the covalent Co‐S interaction, potentially affecting both the electronic dispersion and magnetic exchange interactions.

Furthermore, the DOS reveals a substantial shift of Co 3d‐related states near the VBM by 1.8 eV to higher binding energy (label 2 with an arrow indicating the energetic shift). This shift signals a change in the oxidation state of Co, in agreement with the core‐level shifts observed in the Co 2p XPS spectra. While this effect is also evident in the ARPES data, it is less pronounced than in the theoretical calculations. We attribute the discrepancy to the theoretical assumption of a full electron donated per unit cell, whereas the experimental electron doping level is likely lower. To test this interpretation, we conducted a complementary experiment using cesium instead of lithium. Due to its larger atomic radius, cesium is expected to donate fewer electrons per unit cell. As shown in Figure S9, the CoPS_3_ band structure shows similar qualitative modifications upon Cs doping, but the shift of the peak near the VBM is less pronounced, confirming that the magnitude of the Co‐related band shift scales with the amount of charge transferred, and reinforcing our interpretation of doping‐induced oxidation state changes.

To gain deeper insight into the charge transfer mechanism upon lithium doping, we calculated the charge density difference for the Li‐doped CoPS_3_ system, shown in Figure 4e, using the expression:

$$\Delta \rho =\rho_{\mathrm{Li}/{\mathrm{CoPS}}_{3}}\;-\rho_{{\mathrm{CoPS}}_{3}}-\rho_{\mathrm{Li}}$$

where $\rho_{\mathrm{Li}/{\mathrm{CoPS}}_{3}}$, $\rho_{\mathrm{CoP}S_{3}}$, and $\rho_{\mathrm{Li}}$ denote the charge densities of the Li‐doped system, the pristine CoPS_3_, and an isolated Li atom, respectively. In the resulting plot, yellow and cyan regions represent electron accumulation and depletion, respectively. The data clearly show that the Li atom donates its charge to the CoPS_3_ lattice, with the resulting electron density concentrating primarily around the Co and S atoms. This confirms charge transfer and the interaction between Li and the host lattice. To quantify the amount of transferred charge, we performed a Bader charge analysis. The results show that Li donates approximately 0.89 electrons to the CoPS_3_ lattice. Most of this charge is delocalized over the neighboring Co and S atoms, indicating a strong interaction and redistribution of electron density within the lattice.

**FIGURE 5:**
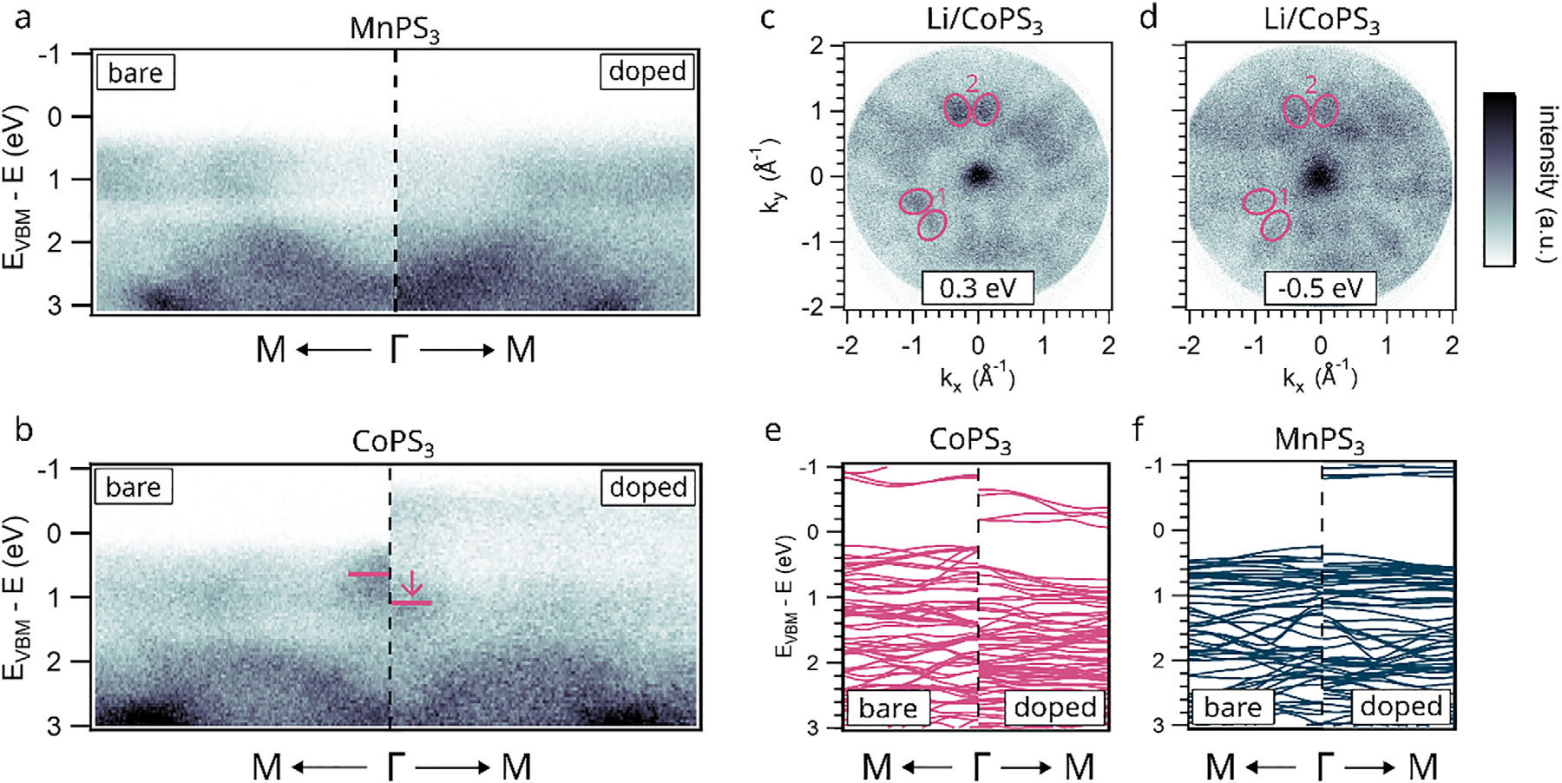
Changes due to alkali metal doping investigated with ARPES. (a) and (b) depict band structure cuts along the M—Γ—M high symmetry direction of the hexagonal BZ for Mn‐ and CoPS_3_, respectively. In contrast to Figure 3, this data set is not symmetrized. The left sides show the signal stemming from the clean surfaces, and the right side shows the signal from the lithium doped surfaces. The purple lines in (b) track the shift of the feature around Γ, indicated also by a small arrow. (c) and (d) display momentum maps for Li/CoPS_3_ at 0.3 eV below and 0.5 eV above the pristine VBM, respectively. Key differences in the momentum dependent intensity are marked by purple ellipsoids (e) and (f) show DFT+U calculations for the clean and Li‐doped CoPS_3_ and MnPS_3_ monolayers, respectively. The calculations show the same induced behavior as extracted from the ARPES measurements with a shift of the bands close to the VBM and new states appearing in the previous bandgap for CoPS_3_, while MnPS_3_ shows only negligible differences, except a slight shift of the VBM.

After identifying the alkali metal‐induced changes using angle‐integrated photoemission and DOS calculations, momentum microscopy (MM) provides further insights into the electronic structure across the entire Brilloiun zone (BZ). We begin by examining the band structure along specific high‐symmetry directions. Figure 5a,b presents ARPES cuts along the M–Γ–M path for the bare and Li‐covered surfaces of MnPS_3_ and CoPS_3_, respectively. As discussed previously, MnPS_3_ (Figure 5a) exhibits negligible changes upon lithium doping, aside from a general broadening of features and a partial closing of the gap around 1.4 eV. The overall band dispersion remains essentially unchanged within the examined energy window. In contrast, lithium doping induces pronounced modifications in the band structure of CoPS_3_ (Figure 5b), the compound with the least stable d‐shell configuration, particularly affecting bands derived from Co 3d orbitals. The spectral features closest to the pristine VBM, which we use as reference energy for all spectra, shift to higher energy by approximately 400 meV, as highlighted by a purple arrow at Γ. In addition, strong spectral weight emerges up to 1 eV above the pristine VBM, with clearly dispersive features visible at both the Γ and M points of the surface BZ. This emergent occupation indicates a transition toward metallic behavior. Complementary cesium doping experiments, confirming that the extent of charge transfer into the d‐shell, and its impact on the band structure, scales with the doping level (see Figure S11).

To understand the nature of the emerging occupation above the pristine VBM, Figure 5c,d displays momentum maps (intensity vs. *k_x_, k_y_*) of Li‐doped CoPS_3_ at two selected energies:

0.3 eV below and 0.5 eV above the pristine VBM. The momentum‐dependent intensity distributions demonstrate clear differences that are indicated by purple ellipsoids in two marked regions. While region 1 only shows a reduced and nearly vanishing intensity of the features for the lithium doped surface, region 2 displays a similar reduction in intensity accompanied by two features arising slightly below in the image to the left and right. These differences together with the still overall similar momentum dependent pattern of both maps confirm the dispersive character of the bands above the pristine VBM, indicating that lithium doping leads to a significant modification of the CoPS_3_ band structure. Based on our previous analysis, we attribute this effect to be primarily driven by changes in the electronic configuration of the Co ion.

Figure 5e shows the DFT+U derived band structures of pristine and lithium doped CoPS_3_. In contrast to the band structure shown in Figure 3, these calculations are based on the full unit cell, resulting in a greater number of visible bands. While pristine CoPS_3_ exhibits semiconducting behavior with a finite bandgap, the introduction of lithium leads to an upward shift of the Fermi level, indicative of n‐type doping. This shift closes the bandgap and introduces bands crossing the Fermi level, suggesting a transition from semiconducting to metallic behavior. The dispersive nature of these bands near the Fermi level agrees well with experimental observations and suggests enhanced electronic conductivity upon doping. In contrast, Figure 5f illustrates the calculated band structure of MnPS_3_ with and without lithium. No significant modifications are observed, apart from a slight rigid shift to higher energy, consistent with both the ARPES and DOS results. This behavior is the consequence of the localization of electrons on ligand orbitals, due to the high stability of its half‐filled 3d^5^ shell. This suggests that charge transfer alone is insufficient to induce substantial electronic reconstruction in systems with electronically rigid d‐shells.

Similar lithium and cesium doping experiments were carried out on NiPS_3_ and FePS_3_. Although XPS measurements indicate oxidation state changes comparable to those seen in CoPS_3_, the ARPES data reveal markedly different electronic responses. In FePS_3_, only very weakly dispersive states appear above the pristine VBM, while in NiPS_3_, a pronounced increase in spectral weight is observed without clear band dispersion. These results suggest that, although charge transfer occurs, it does not induce the formation of dispersing states near the Fermi level as in CoPS_3_. The corresponding EDCs and ARPES spectra for FePS_3_ and NiPS_3_ are provided in Figure S11.

Consistently, DFT+U calculations for Li‐doped FePS_3_ and NiPS_3_ (Figure S13) reveal only minor modifications of the electronic structure, in agreement with previous theoretical work on potassium‐doped NiPS_3_ [26, 38, 39]. These findings show that although all MPS_3_ compounds accept electron doping to some extent, only CoPS_3_ undergoes a significant reorganization of its low‐energy band structure, highlighting its distinct chemical and electronic tunability. Collectively, our theoretical analysis shows how alkali metal doping influences the magnetic properties of these compounds. The calculations reveal a doping‐induced reorganization of the transition‐metal d‐states, particularly in CoPS_3_, which alters the d‐orbital occupation that determines the local magnetic moment. Furthermore, the donated charge is shared between the metal and sulfur atoms, modifying the metal‐ligand hybridization. This directly impacts the superexchange pathways that govern the collective magnetic order [39]. These predictions are consistent with established literature showing that chemical intercalation can induce magnetic phase transitions in MPS_3_ materials [8, 40], underscoring the potential of controlled d‐shell filling as a pathway to engineer magentic order.

## 3 Conclusion

In this work, we combined XPS, ARPES, and DFT+U calculations to investigate how alkali metal doping modulates the electronic structure of the MPS_3_ family of layered antiferromagnets. Our results reveal distinct and compound‐specific doping mechanisms: while Fe‐, Co‐, and NiPS_3_ readily accommodate additional electrons in their transition metal d‐orbitals, MnPS_3_ resists such doping due to the stability of its half‐filled 3d^5^ configuration. Instead, the donated electrons localize in the ligand environment, specifically within the P_2_S_6_ clusters, without significantly altering the electronic band structure near the pristine VBM. This underscores the critical role of the transition metal's electronic configuration in governing charge redistribution.

For Fe‐, Co‐, and NiPS_3_, XPS reveals clear oxidation state changes in the M^2^^+^ ions, indicating d‐orbital occupation upon doping. In CoPS_3_, ARPES measurements show a pronounced restructuring of the valence band, including a shift of Co‐derived bands and the emergence of new dispersive metallic states above the pristine VBM. These modifications—supported by DFT+U calculations and corroborated by complementary Cs doping experiments—demonstrate a doping‐controlled transition from semiconducting to metallic behavior and point toward a potential influence on magnetic exchange interactions.

In contrast, Li doping induces minimal changes in the experimental and theoretical band structures of MnPS_3_, FePS_3_, and NiPS_3_, suggesting that charge transfer alone is insufficient to induce significant electronic reorganization in these systems. Future XMCD measurements will be crucial to elucidate the role of orbital occupation in tuning magnetic order, while time‐resolved ARPES may shed light on the influence of doping on carrier dynamics—particularly in promising candidates like FePS_3_ [12].

Overall, our study establishes alkali metal doping as a powerful, controllable method to modulate the electronic structure of 2D antiferromagnets. The ability to tune band structure and charge distribution in compounds with a less energetically stable d‐shell opens new opportunities for engineering the spin and electronic properties of ionically bonded 2D materials for future spintronic and quantum technologies [2, 41, 42].

## 4 Methods

### 4.1 Sample Preparation

The MPS_3_ crystals were grown via chemical vapor transport and purchased commercially from HQ Graphene. The exfoliation of the (bulk) crystals was carried out using standard scotch‐tape exfoliation in a separate chamber of an ultra‐high vacuum (UHV) system at a base pressure of below 1 × 10^−9^ mbar. Alkali metal deposition was performed using a wire‐shaped dispenser from SAES Industrial, which contains the alkali metal in form of a stable salt combined with a getter material. The deposition was performed at pressures around 1 × 10^−8^ mbar by heating the dispenser with a DC electric current of 8.5 A. Survey spectra of the various samples before and after lithium deposition are reported in Sections S14 and S15.

### 4.2 Momentum Microscopy and X‐ray Photoelectron Spectroscopy

XPS measurements were conducted using a SPECS Phoibos 150 hemispherical analyzer from SPECS GmbH. The X‐ray source employed in the experiments was a monochromatized Al Kα line with a photon energy of 1486.8 eV. The beam spot size on the sample was around 2 mm in diameter. All measurements were carried out using identical source and analyzer settings: a voltage of 12.1 kV and a constant power of 111 W. As the samples investigated are semiconductors, the Fermi edge used to calibrate the binding energy scale was extracted from measurements on an Au(111) single crystal. XPS spectra were analyzed with the XPS Tools (XPST) [43] package for Igor Pro. Peak fitting was based on a Gauss‐Lorentzian sum function to approximate a Voigt profile and incorporates a Shirley background. The Gauss‐Lorentzian ratio was set to 0.3.

The measurements of the valence band structure were taken with the Kreios 150 MM momentum microscope from SPECS GmbH [44, 45], coupled to a UVS 300 UV light source with a monochromator and a fs‐XUV source [38]. For data acquisition, the He 1α line with a photon energy of 21.22 eV and the 10^th^ harmonic of the XUV source at 26.4 eV were used, both with p‐polarization. All data was acquired at room temperature (300 K), corresponding to the paramagnetic phase of the different MPS_3_ compounds. The instrument allows different reciprocal space magnifications, the lowest of which results in an accessible photoelectron parallel momentum of up to ± 3 Å^−1^. For the reported momentum maps, the second lowest k‐magnification was used, enabling a total available parallel momentum of ±2.2 Å^−1^.

### 4.3 Density Functional Theory (DFT)

All DFT [46, 47] calculations were performed for monolayer and bulk crystals using the Vienna Ab initio Simulation Package (VASP) [48, 49]. Ion‐electron interactions for all elemental constituents were described using the projector augmented wave (PAW) method as implemented in VASP. The Perdew–Burke–Ernzerhof (PBE) exchange–correlation functional [50] under the generalized gradient approximation (GGA) was employed. We have performed DFT+U calculations on the monolayer structures for both nonmagnetic (NM) and paramagnetic (PM) phases. The structural optimization was carried out using the PBE+U functional, incorporating the Hubbard U parameters [51] to accurately describe the strongly correlated systems. Empirical U values of 1.9 [11], 4.5, 4.6, and 5.2 eV [52] were used for Fe, Ni, Co, and Mn, respectively. The U values were derived by comparison of the experimentally obtained band structures to calculations for different U values (see Figure S16). Self‐consistent calculations were performed with PBE+U along with van der Waals (vdW) corrections, relaxing all ions until the Hellmann–Feynman forces were less than 0.001 eV/Å. The two‐body vdW interaction, as devised by Tkatchenko–Scheffler, was included during optimization. A kinetic energy cut‐off of 600 eV was used for the plane‐wave basis set and the electronic self‐consistency loop was converged to within 10^−5^ eV. A Γ‐centered 6 × 6 × 6 k‐point grid was used to sample the Brillouin zone, unless stated otherwise. For monolayer calculations, a vacuum spacing of 15 Å was added to eliminate spurious interactions between periodic images. In the NM calculations, we neglected spin degrees of freedom and performed non‐spin polarized DFT calculations [53, 54, 55]. The PM phase was mimicked using a 4 × 4 supercell with randomly oriented spins, resulting in zero net magnetization, generated via the special quasirandom structures (SQS) approach [56]. While methods such as DFT+DMFT can provide a more accurate and theoretically justified description of the paramagnetic state of magnetic materials [51], they are computationally intensive and beyond the scope of this broad materials survey. Furthermore, recent studies on FePS_3_ and MnPS_3_ show that DFT+U band structures agree well with ARPES in both magnetic and paramagnetic phases [13, 14, 52]. Crucially, Lazić et al. concluded that “a mean‐field DFT+U approach is sufficient to effectively reproduce the experimental photoemission spectra” for this class of materials [53]. The comparison of bulk and monolayer band structures is given in Figure S17. We have also plotted the band structures for the AFM phase of all compounds (see Figure S18). Band unfolding has been performed using the BandUp code [57]. All calculated and experimental band structures are aligned to the valence band maximum (VBM). The slight offset of the topmost valence band from exactly zero energy arises because the VBM does not necessarily lie on the plotted high‐symmetry path and due to finite k‐point sampling, unfolding, and experimental broadening.

### 4.4 Formation Energy

The crystal structures of all transition metal phosphorus trichalcogenides (MPS_3_; M = Fe, Ni, Co, Mn) were taken from experimental data in the paramagnetic phase. These compounds crystallize in a monoclinic layered structure with the C2/m space group (No. 12). The Li atom was doped into the 2 × 2 × 2 supercells at different sites and optimized using the PBE+U functional. The most stable geometry was considered where the Li atom is intercalated between the layers. Subsequently, to assess the stability of these compounds, the formation energies [58] were calculated using the following equation:

$$E_{f}=E_{defect}\;-\;E_{pristine}\;-\;n_{Li}\mu_{Li}$$

where *E_f_*, *E_defect_*, *E_pristine_*, *n_Li_* and µ_*Li*_ represent the defect formation energy, total DFT energy of the doped system, DFT energy of the pristine system, number of Li atoms, and the chemical potential of Li, respectively. The computed formation energies of FePS_3_, NiPS_3_, CoPS_3_ and MnPS_3_ are −1.39, −2.07, −3.71, and −1.49 eV, respectively, suggesting that all these compounds are thermodynamically stable.

## Author Contributions

Conceptualization, J.E.N., M.C.; Methodology, J.E.N., and P.B.; Experimental investigation, J.E.N., T.W., P.M., and M.G.; Experimental support, D.M.J., L.S., K.S., M.S.A., V.M., and M.Ca.; Data analysis. J.E.N. and T.W.; Theoretical calculations, P.B. and S.B.; Writing – original draft, J.E.N., P.B., and M.C.; Writing – review and editing, J.E.N., P.B., T.W., P.M., D.M.J., L.S., K.S., V.M., M.S.A., S.B., and M.C.; Funding acquisition, S.B. and M.C.

## Conflicts of Interest

The authors declare no conflicts of interest.

## Declaration of generative AI and AI‐assisted technologies

During the preparation of this work, the authors used ChatGPT to enhance the quality of writing by improving grammar, style, and clarity. After using this tool/service, the authors reviewed and edited the content as needed and take full responsibility for the content of the publication.

## Supporting information




**Supporting File**: advs74677‐sup‐0001‐SuppMat.docx.

## Data Availability

The data that support the findings of this study are available from the corresponding author upon reasonable request.
