# Supplementary material for: Electronic Structure Reorganization in MPS3 via d‐Shell‐Selective Alkali Metal Doping
Source: Adv Sci (Weinh). 2026 Mar 24;13(27):e10675. doi: 10.1002/advs.202510675 (PMC13170249; doi:10.1002/advs.202510675)
Supplement: Supplementary file 1 — Supporting File: advs74677‐sup‐0001‐SuppMat.docx. [file ADVS-13-e10675-s001.docx]

**Supplementary Information: Electronic Structure Reorganization in MPS₃ via d-Shell-Selective Alkali Metal Doping**

Jonah Elias Nitschke^1*^, Preeti Bhumla^2^, Till Willershausen^1^, Patrick Merisescu^3^, David Maximilian Janas^1^, Lasse Sternemann^1^, Michael Gutnikov^1^, Karl Schiller^1^, Valentin Mischke^1^, Michele Capra^1^, Mira Sophie Arndt^1^, Silvana Botti^2^, Mirko Cinchetti^1**^

^1^ TU Dortmund University, Otto-Hahn-Straße 4, 44227 Dortmund, Germany

^2^ Research Center Future Energy Materials and Systems of the University Alliance Ruhr and Interdisciplinary Centre for Advanced Materials Simulation, Faculty of Physics and Astronomy, Ruhr University Bochum, Universitätsstraße 150, D-44801 Bochum, Germany

^3^ University of Bath, Clavertown Down, Bath BA2 7AY, United Kingdom

*E-mail: jonah.nitschke@tu-dortmund.de

**E-mail: [mirko.cinchetti@tu-dortmund.de](mailto:mirko.cinchetti@tu-dortmund.de)

**Supplementary Information**

1. **M 2p multiplet fit for Mn- and CoPS_3_**


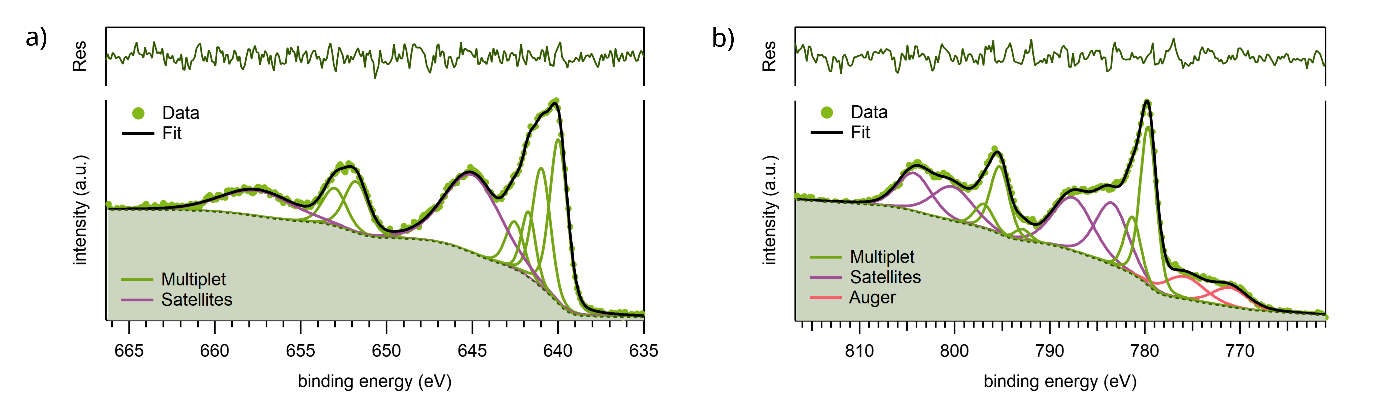


**Figure S1.** **XPS spectra of the M 2p peaks for MnPS_3_ and CoPS_3_.** XPS spectrum of the (a) Mn 2p peak and (b) Co 2p peak. The fits consist of the 2p_3/2_ and 2p_1/2_p peaks including their multiplet structure (green curves, based on the Literature by Gupta et al.) and up to two satellites (violet curves). Additionally, the spectrum for CoPS_3_ shows an additional shoulder at lower binding energies stemming from the L_2_M_23_M_45_ Auger transition (red curves). The fit parameters (position and FWHM) are given in Table S1 below.

**Table S1. Extracted fit parameters for the M 2p peaks of Mn- and CoPS_3_.**

| **Material** | **Peak** | **Position /eV** | **FWHM / eV** | **Type** |
| --- | --- | --- | --- | --- |
|  |  |  |  |  |
| **MnPS_3_** | 1 | 639.9 | 1.2 | Multiplet |
|  | 2 | 640.9 | 1.2 | Multiplet |
|  | 3 | 641.7 | 0.9 | Multiplet |
|  | 4 | 642.5 | 1.2 | Multiplet |
|  | 5 | 644.9 | 3.8 | Satellite |
|  | 6 | 651.7 | 1.6 | Multiplet |
|  | 7 | 653.0 | 1.6 | Multiplet |
|  | 8 | 657.5 | 4.9 | Satellite |
|  |  |  |  |  |
| **CoPS_3_** | 1 | 771.0 | 4.4 | Auger |
|  | 2 | 775.8 | 5.0 | Auger |
|  | 3 | 779.6 | 1.9 | Multiplet |
|  | 4 | 781.2 | 1.9 | Multiplet |
|  | 5 | 783.4 | 4.0 | Satellite |
|  | 6 | 787.5 | 5.2 | Satellite |
|  | 7 | 792.8 | 1.9 | Multiplet |
|  | 8 | 795.2 | 2.3 | Multiplet |
|  | 9 | 796.9 | 2.3 | Multiplet |
|  | 10 | 800.2 | 5.1 | Satellite |
|  | 11 | 804.2 | 4.2 | Satellite |

1. **Evolution of M 2p peaks under stepwise doping with lithium**


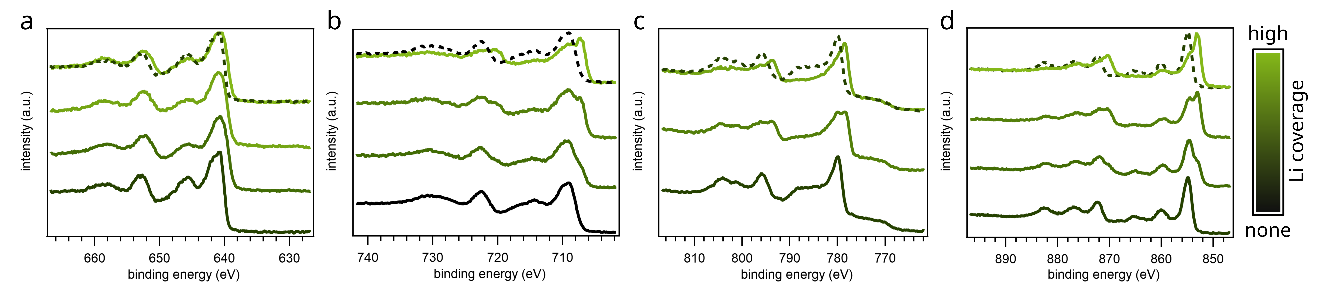


**Figure S2. Evolution of M 2p spectra under stepwise intercalation with Li atoms.** As mentioned in the main text, for MnPS_3_ (a), no clear changes in the signal besides a slight broadening were observed. In contrast, FePS_3_ (b), CoPS_3_ (c), and NiPS_3_ (d) undergo strong modifications. In all cases, the deposition of lithium leads to a stepwise increase in the occurrence of additional peaks at lower binding energies with respect to the 2p_3/2_ and 2p_1/2_ peaks.

1. **Change in M 2p splitting**

**Table S3.** Extracted shifts for the two split 2p peaks of the different transition metal ions.

| **Compound** | **2p_3/2_** | **Shift** | **2p_1/2_** | **Shift** | **Δ_Doped_** | **Δ_Bare_** |
| --- | --- | --- | --- | --- | --- | --- |
| FePS_3_ | 707.2 | - 1.7 | 720.2 | - 2.1 | 13 | 13.4 |
| CoPS_3_ | 778.4 | - 1.3 | 793.4 | - 2.3 | 15 | 16 |
| NiPS_3_ | 853.2 | - 1.1 | 870.3 | - 1.3 | 17.1 | 17.3 |

1. **Binding energies of main features for the M 2p spectra**

**Table S4.** **Energetic positions of the different spectral features in M 2p spectra.** Extracted binding energies for the 2p peaks of the transition metal ions, as well as the energetic separations of their satellite features for Mn-, Fe-, Co- and NiPS_3_. All values are given in binding energy. All extracted peak positions from the fits are provided in SI sections S1 and S5.

|  | 2p_3/2_ | Sat1_3/2_ | Sat2_3/2_ | 2p_1/2_ | Sat1_1/2_ | Sat2_1/2_ |
| --- | --- | --- | --- | --- | --- | --- |
| Material | E / eV | ΔE / eV | | E / eV | ΔE / eV | |
| MnPS_3_ | **639.9** | **+ 5.0** | **-** | **651.7** | **+ 5.8** | **-** |
| FePS_3_ | **708.5** | **+ 5.3** | **-** | **721.7** | **+ 6.6** | **-** |
| CoPS_3_ | **779.6** | **+ 3.8** | **+ 6.9** | **795.2** | **+ 5.0** | **+ 9.0** |
| NiPS_3_ | **854.2** | **+ 5.1** | **+ 9.9** | **871.5** | **+ 4.6** | **+ 10.1** |

1. **M 2p multiplet fit for Ni- and FePS_3_**


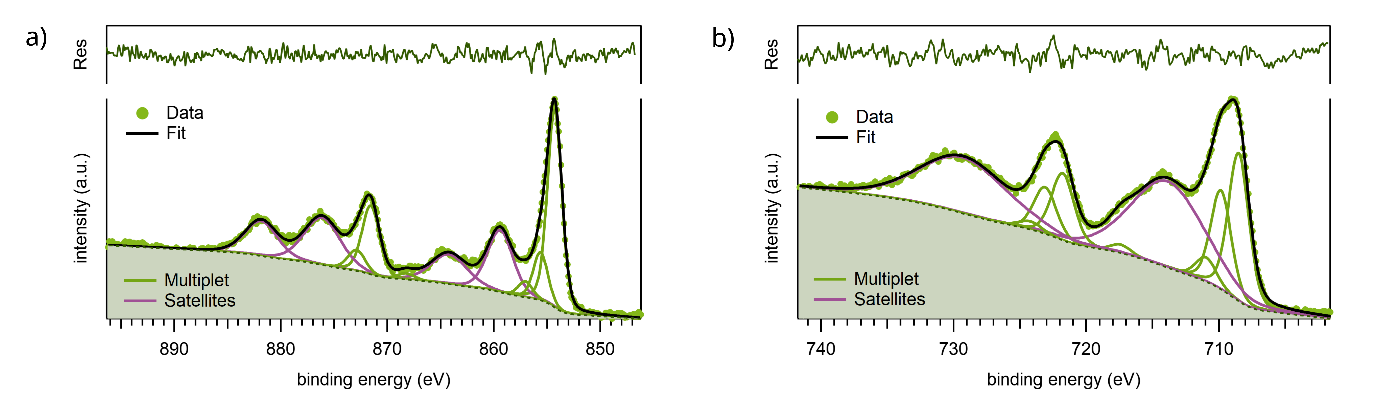


**Figure S5. XPS spectra of the M 2p peaks for NiPS_3_ and FePS_3_.** XPS spectrum of the (a) Ni 2p peak and (b) Fe 2p peak. The fits consist of the 2p_3/2_ and 2p_1/2_ peaks including their multiplet structure (green curves, based on the Literature by Gupta et al) and up to two satellites (violet curves). The fit parameters (position and FWHM) are given in Table S5 below.

**Table S5. Extracted fit parameters for the M 2p peaks of Ni- and FePS_3_.**

| **Material** | **Peak** | **Position /eV** | **FWHM / eV** | **Type** |
| --- | --- | --- | --- | --- |
|  |  |  |  |  |
| **NiPS_3_** | 1 | 854.2 | 1.6 | Multiplet |
|  | 2 | 855.6 | 1.6 | Multiplet |
|  | 3 | 857.0 | 1.6 | Multiplet |
|  | 4 | 859.3 | 2.8 | Satellite |
|  | 5 | 864.1 | 4.1 | Satellite |
|  | 6 | 868.2 | 1.9 | Multiplet |
|  | 7 | 871.5 | 1.9 | Multiplet |
|  | 8 | 872.8 | 1.9 | Multiplet |
|  | 9 | 876.1 | 4.1 | Satellite |
|  | 10 | 881.6 | 3.6 | Satellite |
|  |  |  |  |  |
| **FePS_3_** | 1 | 708.5 | 1.8 | Multiplet |
|  | 2 | 709.8 | 1.8 | Multiplet |
|  | 3 | 710.9 | 1.8 | Multiplet |
|  | 4 | 713.8 | 6.5 | Satellite |
|  | 5 | 717.4 | 1.8 | Multiplet |
|  | 6 | 721.7 | 2.0 | Multiplet |
|  | 7 | 723.1 | 2.0 | Multiplet |
|  | 8 | 724.2 | 2.0 | Multiplet |
|  | 9 | 729.3 | 7.8 | Satellite |

1. **P 2p and S 2p fits for all four MPS_3_**


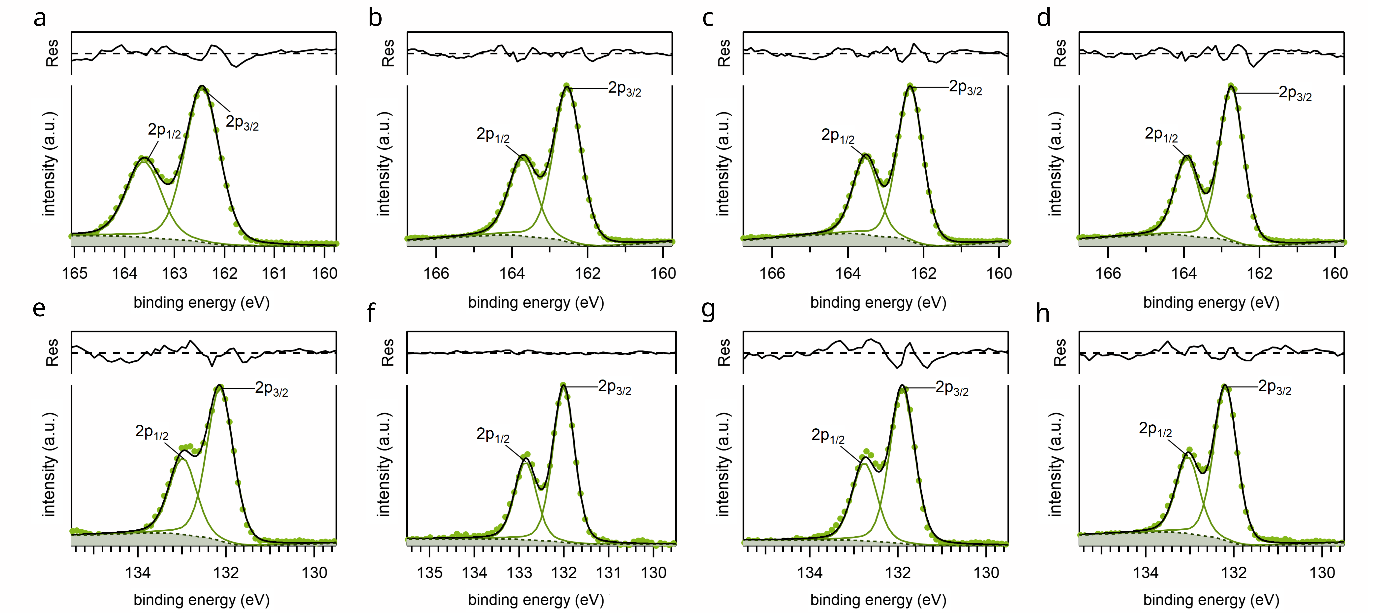


**Figure S6. XPS spectra of S 2p (top row) and P 2p peaks (bottom row).** (a) to (d) show the doublet fitted S 2p peaks for Mn-,Fe-, Co- and NiPS_3_ (from left to right) with the split 2p_3/2_ and 2p_1/2_ peaks. The results from the fit (position and FWHM) are listed in Table S6 below.

**Table S6.** **Extracted fit parameters for the S and P 2p peaks of Mn-, Fe-, Co-, and NiPS_3_.**

| **Material** | **Peak** | **Position /eV** | **FWHM / eV** |
| --- | --- | --- | --- |
|  |  |  |  |
| **MnPS_3_** | P 2p_3/2_ | 132.1 | 0.7 |
|  | P 2p_1/2_ | 132.9 | 0.7 |
|  | S 2p_3/2_ | 162.4 | 0.8 |
|  | S 2p_1/2_ | 163.6 | 0.8 |
|  |  |  |  |
| **FePS_3_** | P 2p_3/2_ | 132.0 | 0.5 |
|  | P 2p_1/2_ | 132.8 | 0.5 |
|  | S 2p_3/2_ | 162.5 | 0.8 |
|  | S 2p_1/2_ | 163.7 | 0.8 |
|  |  |  |  |
| **CoPS_3_** | P 2p_3/2_ | 131.9 | 0.6 |
|  | P 2p_1/2_ | 132.7 | 0.6 |
|  | S 2p_3/2_ | 162.3 | 0.7 |
|  | S 2p_1/2_ | 163.5 | 0.7 |
|  |  |  |  |
| **NiPS_3_** | P 2p_3/2_ | 132.1 | 0.6 |
|  | P 2p_1/2_ | 133.0 | 0.6 |
|  | S 2p_3/2_ | 162.7 | 0.7 |
|  | S 2p_1/2_ | 163.9 | 0.7 |

**Figure S6. XPS spectra of S 2p (top row) and P 2p peaks (bottom row).** (a) to (d) show the doublet fitted S 2p peaks for Mn-,Fe-, Co- and NiPS_3_ (from left to right) with the split 2p_3/2_ and 2p_1/2_ peaks. The results from the fit (position and FWHM) are listed in Table S6 below.

1. **Symmetrization for band structure extraction**


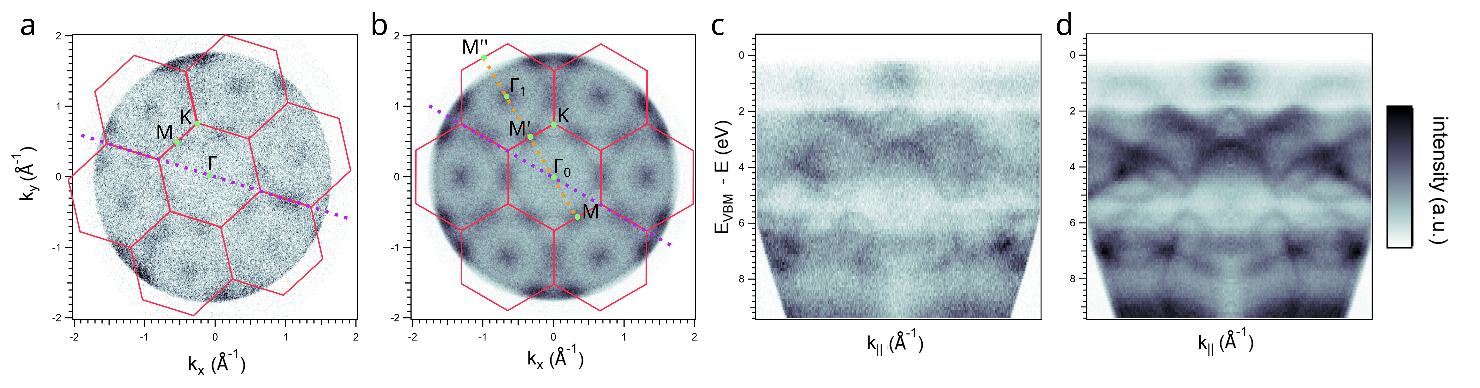


**Figure S7. Symmetrization procedure.** (a) and (b) show the momentum maps extracted from the raw data stack and the symmetrized data stack, respectively. The red hexagonal structure marks the center of the Brillouin zone (BZ) and the surrounding ones, with one pair of K and M points marked as examples . The used photon energy of 21.2 eV provides access to the entire 1^st^ and neighboring Brillouin zones. The purple dashed line marks the cut used for the band structure comparison in (c) and (d). The orange line in (b) marks the M – Γ_0_ – M´ – Γ_1_ – M´´ path used for the comparison of the signal in the different BZs. (c) and (d) present the band structure cuts extracted from the raw and symmetrized data, respectively. By symmetrizing the measured data, we can eradicate the visual influence of the experimental geometry regarding the incident direction of the incoming light. However, this is just a visual adjustment for easier comparison to the DFT+U calculations, as these do not include the experimental geometry. The Symmetrization is performed for each energy separately by summing up the respective maps with are rotated and mirrored copies matching the 6-fold symmetry of the hexagonal Brillouin zone.

1. **A zoomed-in view of the band structures**

**
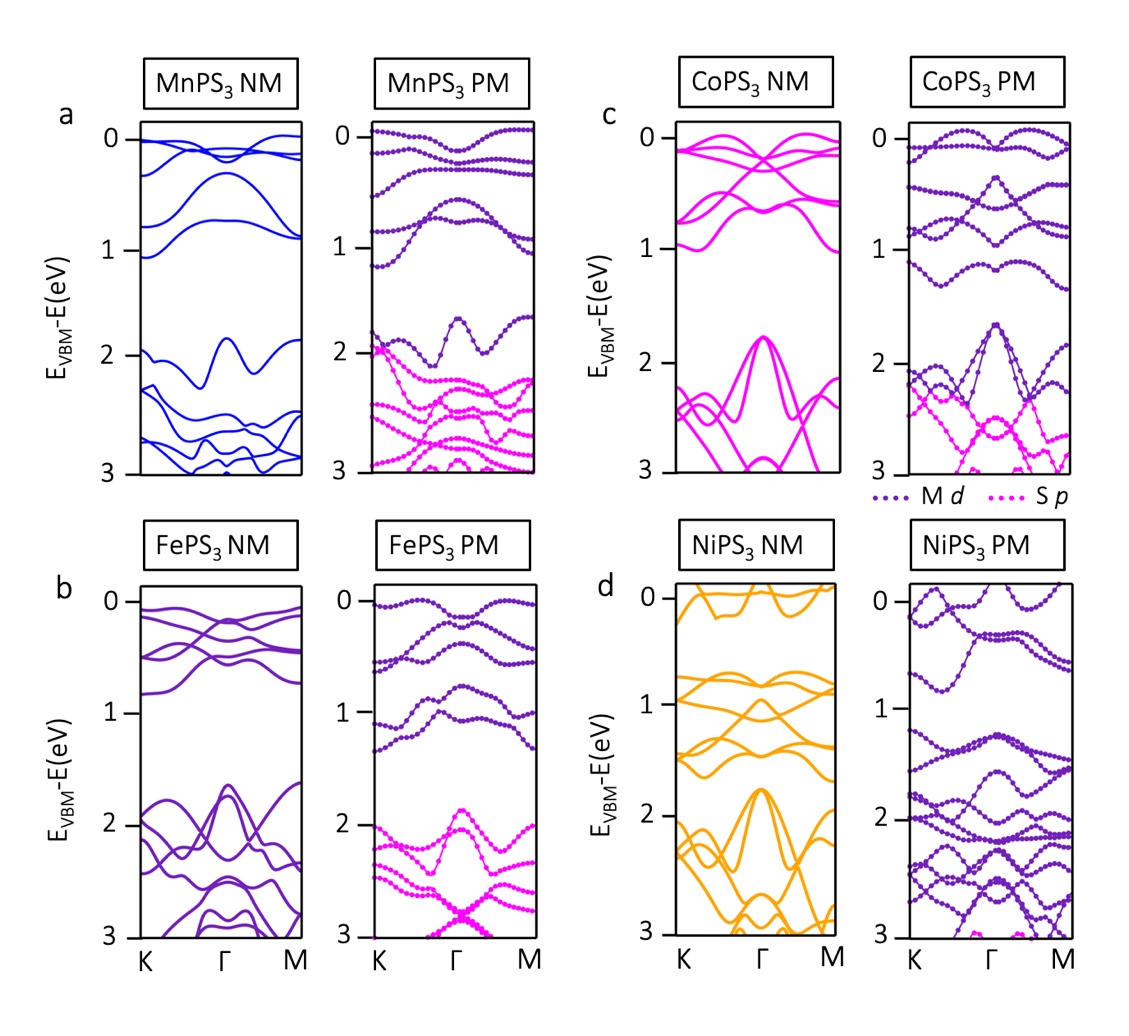
**

**Figure S8.** **DFT+U derived band structures of a) Mn-, b) Fe-, c) Co-, d) NiPS_3_ monolayers along high symmetry path for both NM and PM phases, respectively. The different colors in PM band structures correspond to different orbitals as shown in the figure.**

1. **ARPES measurements of neighboring Brillouin zones**

In addition to the comparison of the central BZ, the high photon energy of 21.2 eV used in the ARPES measurements also enables access to electronic states in neighboring Brillouin zones (BZs), presented in **Figure S8**. As expected, the observable parallel momentum (k_||_) range, commonly referred to as the photoemission horizon, decreases with increasing binding energy due to the reduced kinetic energy of the photoelectrons.

In all four band structures, the data is cut along the Gamma points of the central and neighboring Brillouin zones (Γ_0_ and Γ_1_, respectively), specifically along the path M – Γ_0_ – M´ – Γ_1_ – M´´ (see **Figure S7**). Notable differences in band dispersion between the two zones are observed, particularly around 3 eV and 5 eV below the valence band maximum (VBM). This effect is well known and attributed to the photoemission matrix element^[38]^, which affects the band-specific photoemission signal strength in the first and neighboring BZs based on experimental geometry. Displaying the data from both BZs therefore facilitates a more comprehensive comparison with theoretical prediction.


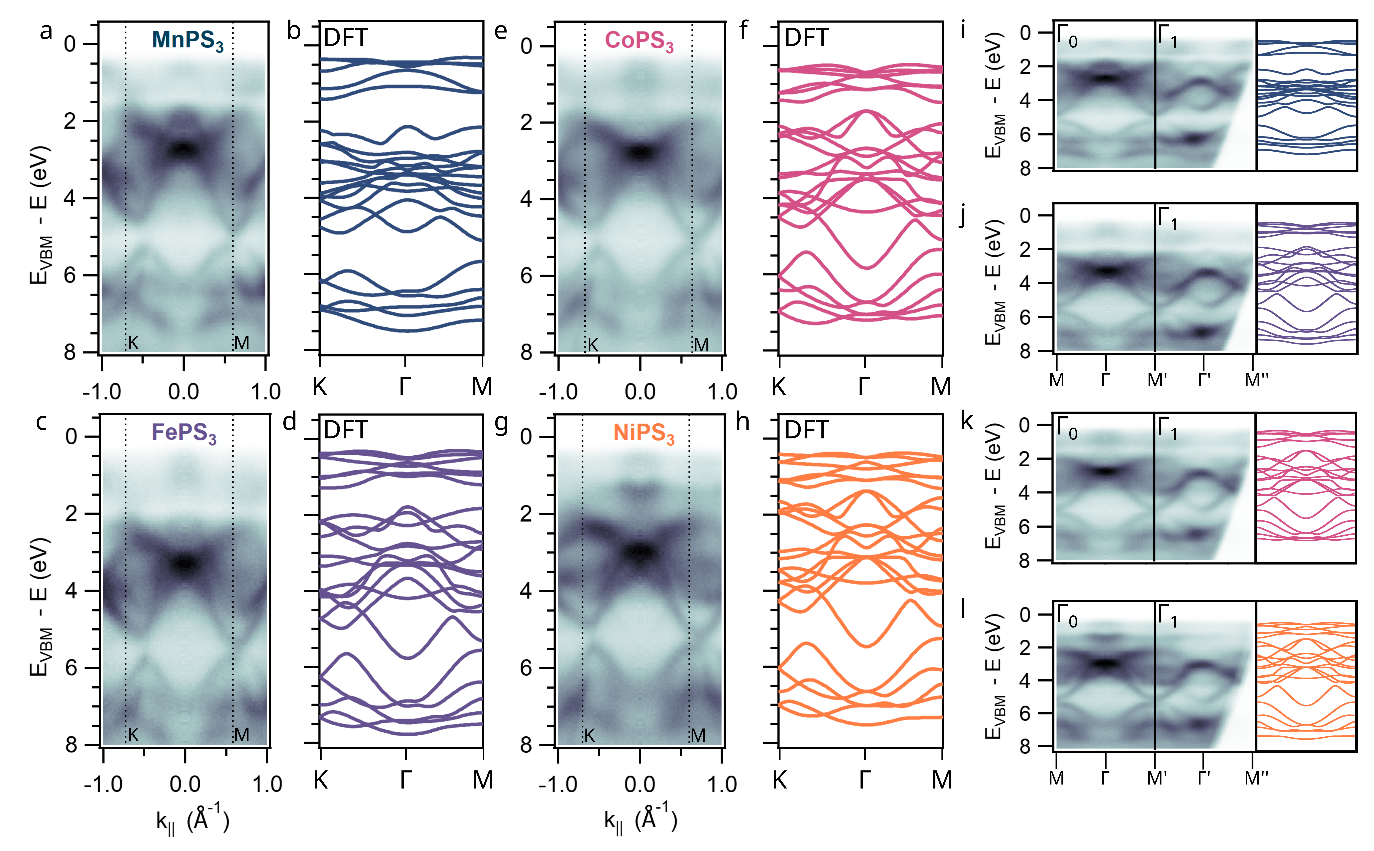


**Figure S9. (i) – (iv) present a comparison of the ARPES intensity in the 1st Brillouin zone (Γ0) and the neighboring BZ (Γ1) together with DFT derived band structures cut along M – Γ – M’ path.**

1. **
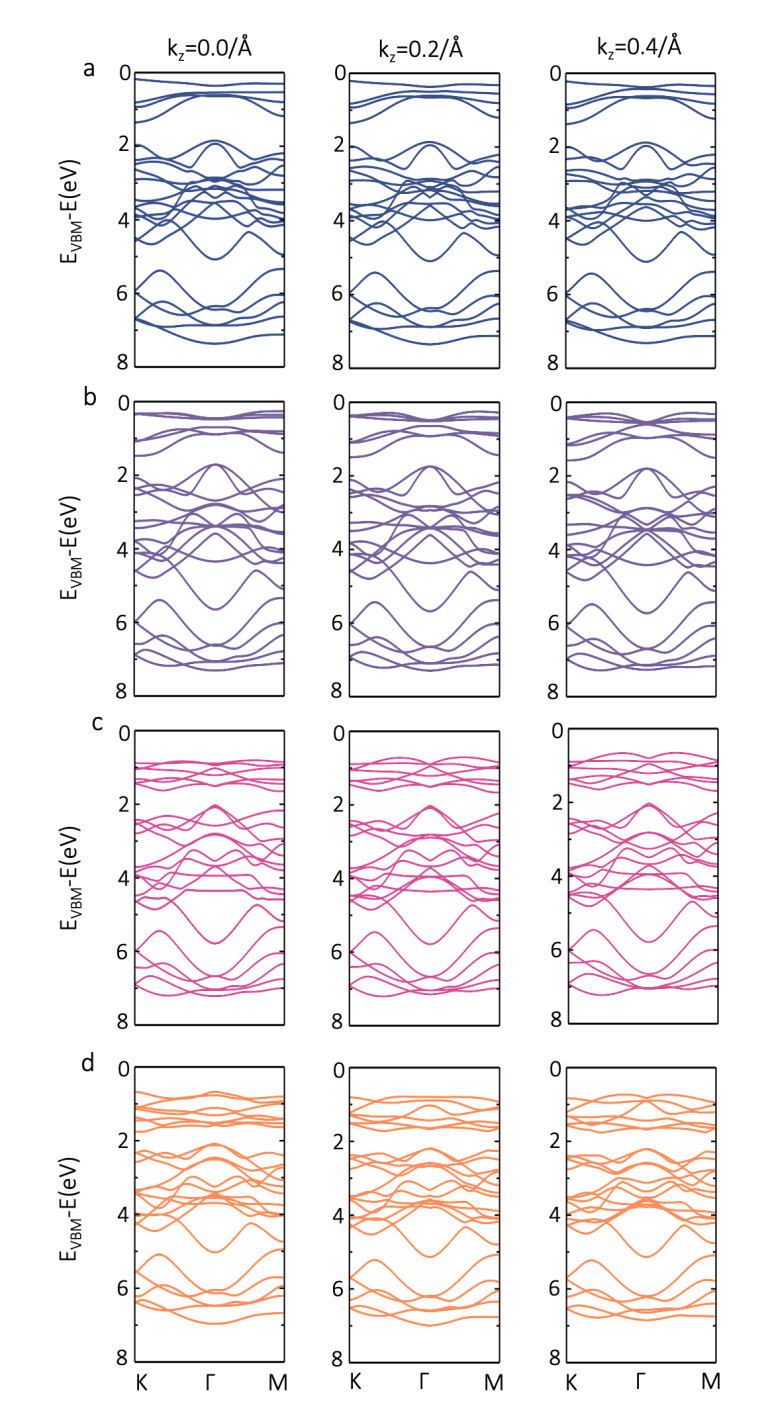
K_z_ dispersion plots**

**Figure S10.** **DFT+U derived bulk band structures in NM phase of a) Mn-, b) Fe-, c) Co-, d) NiPS_3_ along high symmetry path at various k_z_ as marked on top.**

1. **Cs doping of CoPS_3_**


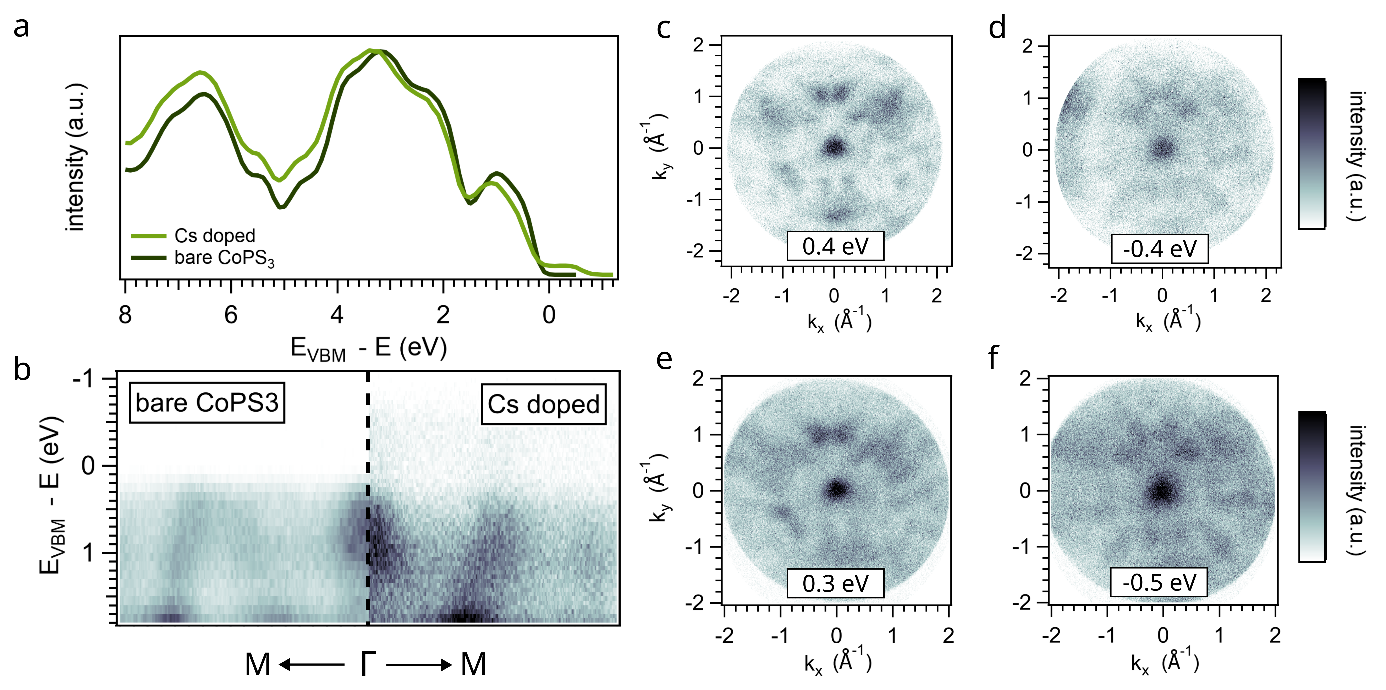


**Figure S11. Cs doping of CoPS_3_.** (a) EDC of CoPS_3_ before (dark green) and after doping with cesium (bright green). While no strong modification of the valence band structure itself is visible, a noticeable increase in intensity above the previous valence band maximum appears. (b) angle-resolved band structure for the bare (left side) and the Cs doped surface (right side) along the M – Γ – M direction. While the feature at around 1.8 eV shows no shift in energy, a small shift is apparent for the features around the VBM. Here, the midpoint in energy of the intensity distribution around Γ shows a slight shift of around 100 meV to higher binding energies. Additionally, new signal above the previous VBM is present up to -0.7 eV. (c) and (d) show momentum maps extracted at 400 meV below and above the previous VBM, respectively. A comparison to the momentum maps extracted for Li deposited on CoPS_3_ (e) and (f) shows that the features visible in momentum space are very similar, especially for the momentum maps acquired above the previous VBM.

1. **
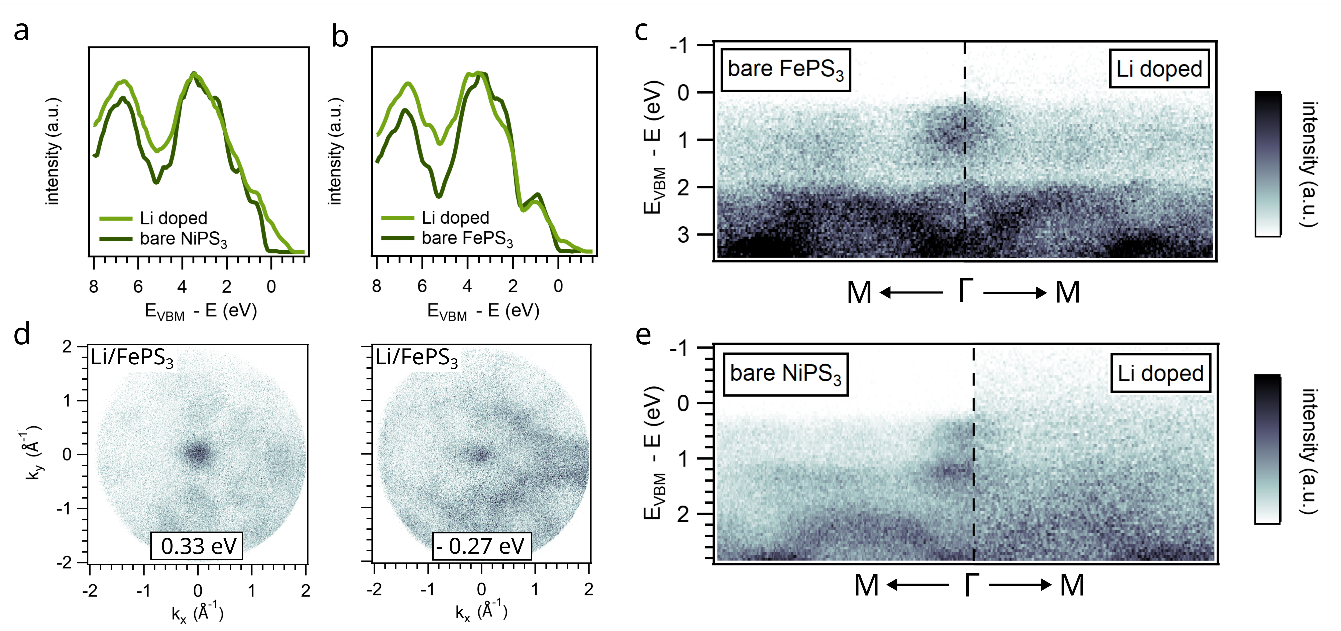
ARPES measurements on FePS_3_ and NiPS_3_**

**Figure S12. Li intercalation of FePS_3_ and NiPS_3_.** (a) and (b) show the changes in the valence band structure induced by Li intercalation of NiPS_3_ and FePS_3_, respectively. In both cases, additional signal above the previous VBM is observed, extending up to -1 eV. (c) shows the band structure of bare and Li intercalated FePS_3_ along the M – Γ – M path, which does not show any modification apart from a slight increase in intensity above the previous VBM. (d) presents momentum maps extracted from lithium doped FePS3 at 0.33 and -0.27 eV binding energy. As one can see, the signal above the previous VBM (right side) contains a clear momentum dependent pattern, different from the occupied states shown on the left side. In contrast, the extracted band structure for Li intercalated NiPS_3_ in (e) shows a strong decrease in the band sharpness, with no momentum dependent pattern being visible in the signal above the previous VBM.

1. **DFT+U calculations for lithium doping on Fe- and NiPS_3_**


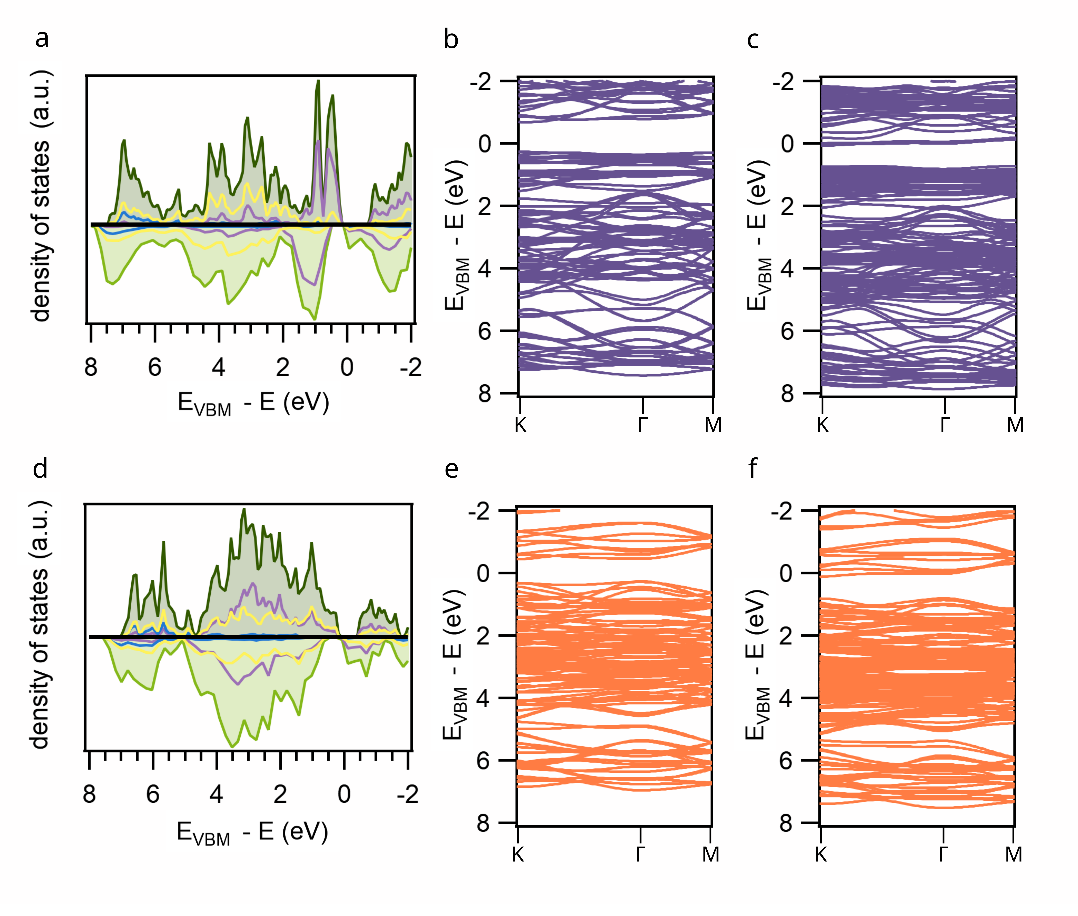


**Figure S13. DFT+U calculations for the PDOS and band structure of Fe- and NiPS_3_ after lithium doping.** (a) PDOS for bare FePS_3_ (top) and lithium doped FePS_3_ (bottom). No drastic changes are apparent besides a slight broadening and a shift of the Fermi energy (visible as a shift of the spectrum), both of which are also observed in the calculated band structures (b, c). display the calculated band structure for bare and lithium doped FePS_3_ based on the bigger unit cell. No strong modifications are apparend. Similar results are also qualitatively obtained from the calculations for NiPS_3_ (d-f).

1. **M 3p spectra for bare and lithium doped surfaces**


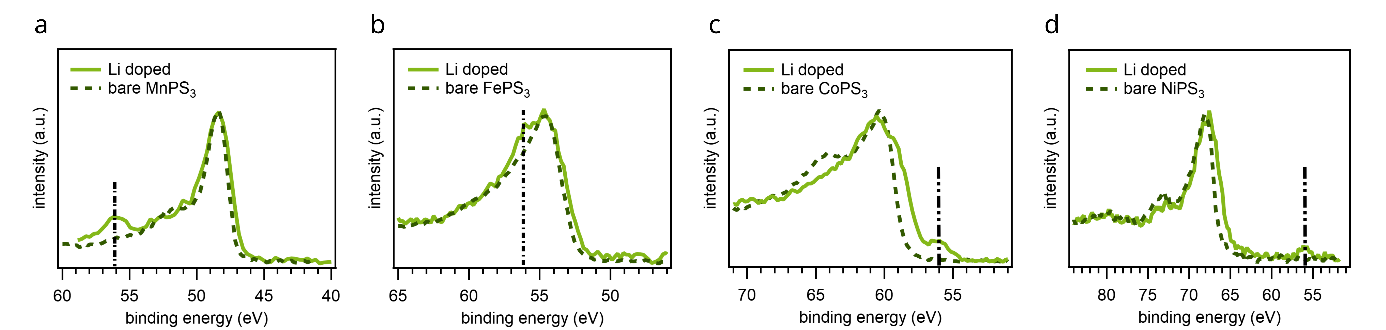


**Figure S14. XPS spectra of the M 3p peak for bare and lithium doped MPS_3_ surfaces.** The spectra show the signal from the bare surface (dark green, dashed) and the Li doped surface (bright green). The Li 1s peak is marked by the vertical dashed line at 56.1 eV binding energy and is noticeable for all four compounds.

1. **Survey spectra with alkali metal doping**


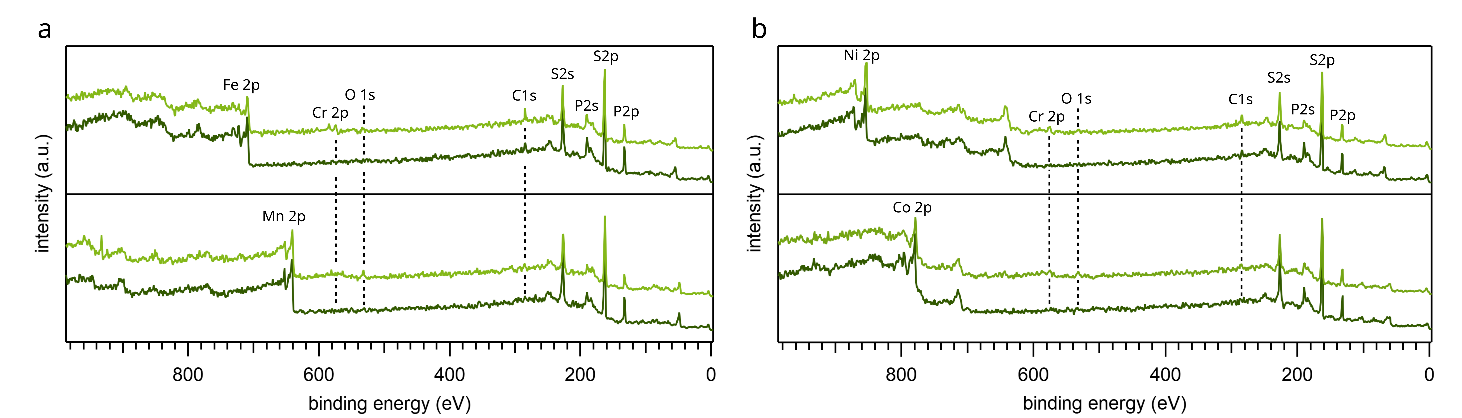


**Figure S15. XPS survey spectra for alkali metal deposition on different MPS**_3_**.** (a) and (b) show the survey spectra obtained for the bare MPS3 surfaces (dark green) and the Li doped surfaces (bright green). The contribution from the different atomic orbitals are marked in the top panel. The core-level peaks corresponding to phosphorus and sulfur atoms appear in the 100–250 eV binding energy range, and their positions remain consistent across all transition metal species, indicating no significant chemical shift. In contrast, the peaks associated with the transition metals are found in two distinct regions: 0–100 eV and above 600 eV, the latter corresponding to the 2p core levels (labeled for each MPS₃ compound). The absence of discernible carbon 1s and oxygen 1s peaks in the spectra confirms the cleanliness of the cleaved crystals as well as after doping. As visible, especially in the survey spectra for FePS_3_, longer usage of the described alkali metal containers lead to the deposition of substantial amounts of chromium. Since FePS_3_ was the last material investigated, the chromium contamination is the highest, while for CoPS_3_ and NiPS_3_, no traces of Cr are visible and for MnPS_3_, only a substantially lower amount is present. For analysis of the different peaks described in the main text, only the spectra with negligible amounts of chromium were used.

1. **Band structure for different U_eff_ values**


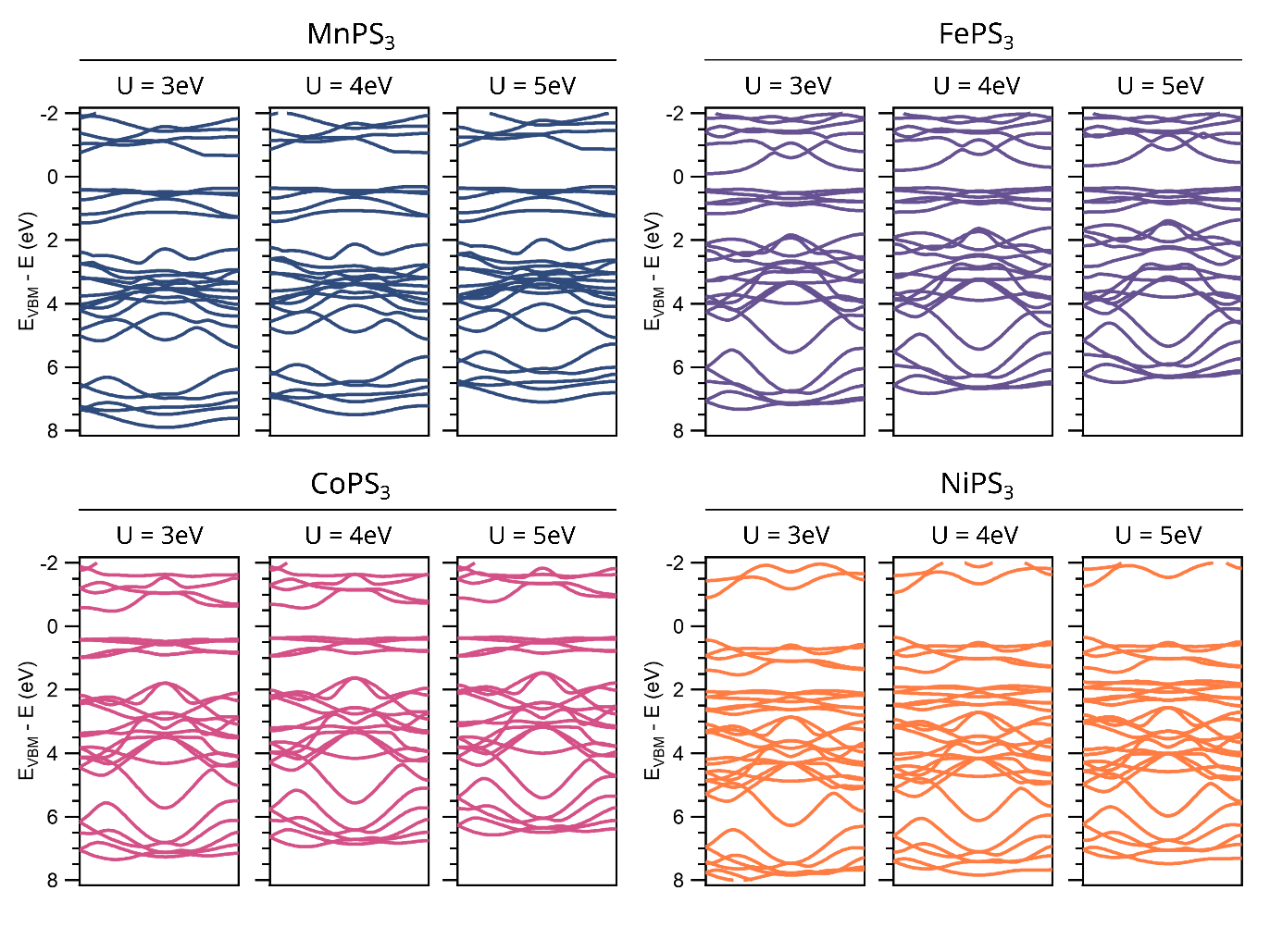


**Figure S16.** **DFT+U derived band structures for different values of U_eff_.** As shown for all four MPS_3_ materials, an increase in the effective U leads to a compression of the bands up to 8 eV binding energy and a bigger gap between the top of the valence band and the bottom of the conduction band. For the calculations presented throughout the paper, the value of U_eff_ was chosen based on the best fit to the experimentally obtained band structure.

1. **Comparison of bulk and monolayer band structures**

**
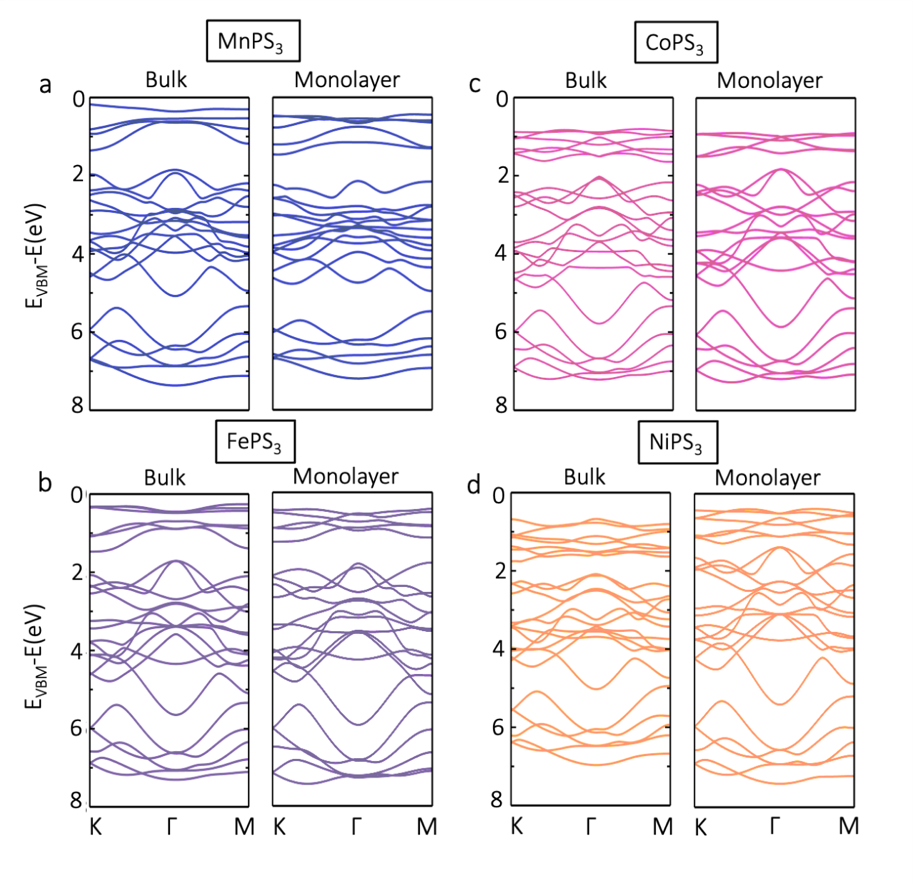
**

**Figure S17.** **DFT+U derived band structures in NM phase of a) Mn-, b) Fe-, c) Co-, d) NiPS_3_ along high symmetry path for bulk and monolayer structures, respectively.**

1. **Comparison of presented DFT+U calculations with the antiferromagnetic ordering**

**
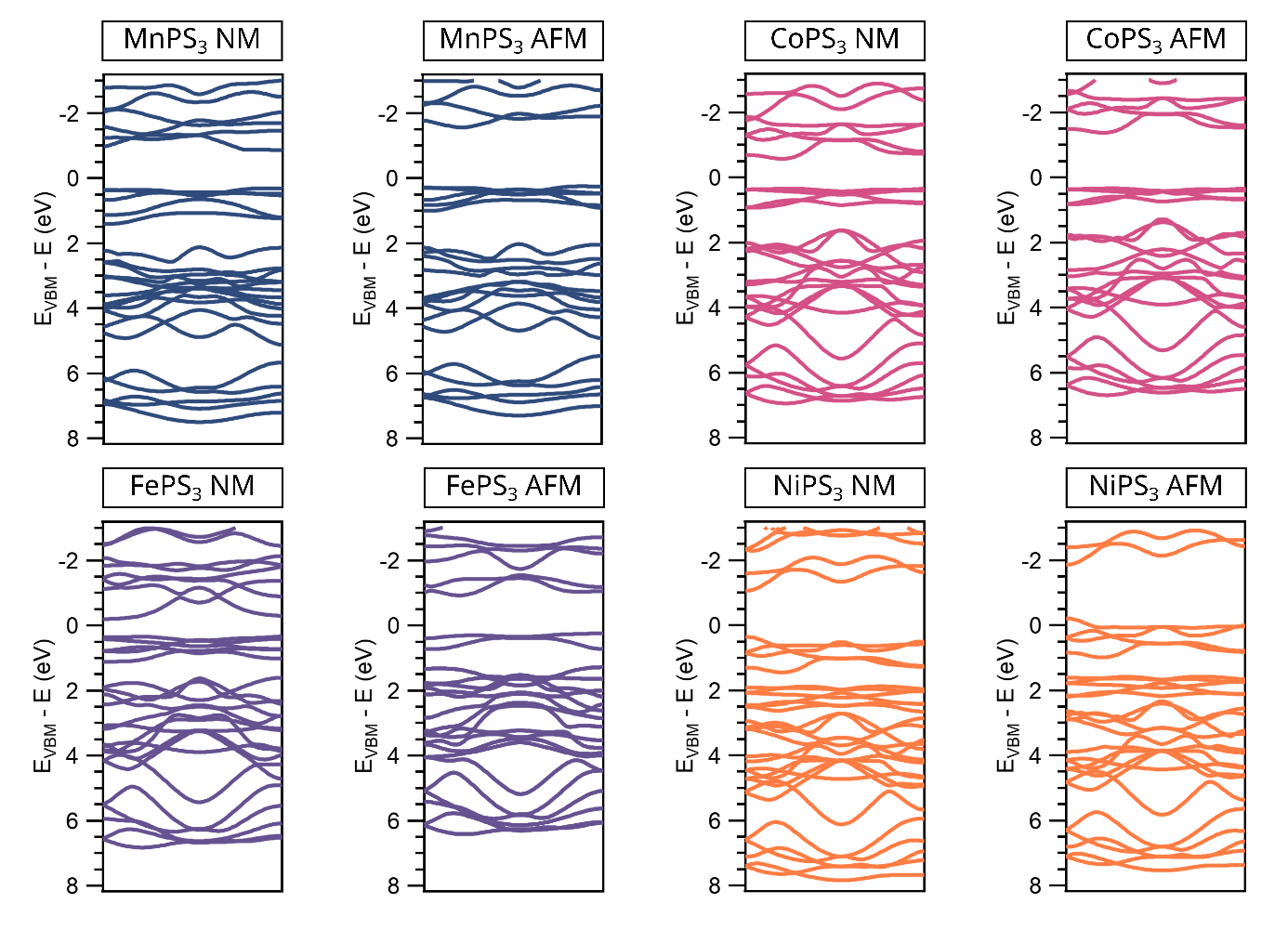
**

**Figure S18.** **Electronic band structures of the different MPS_3_ materials along the K – Γ – M path in the non-magnetic (NM) and antiferromagnetic (AFM) ordering.** These calculations show that introducing magnetic order opens the gap slightly and shifts band energies by a few hundred meV, but the overall band dispersions and orbital characters remain essentially unchanged compared to the non-magnetic case.
